# ReMindCare, an app for daily clinical practice in patients with first episode psychosis: A pragmatic real‐world study protocol

**DOI:** 10.1111/eip.12960

**Published:** 2020-04-06

**Authors:** Lucia Bonet, John Torous, David Arce, Ignacio Blanquer, Julio Sanjuán

**Affiliations:** ^1^ Department of Clinic Medicine, School of Medicine University of Valencia Valencia Spain; ^2^ Division of Digital Psychiatry, Department of Psychiatry, Beth Israel Deaconess Medical Center Harvard Medical School Boston Massachusetts; ^3^ Institute of Instrumentation for Molecular Imaging (I3M) Joint Centre CSIC & Universitat Politècnica de València Valencia Spain; ^4^ Centre of Biomedical Investigation in Mental Health (CIBERSAM) Spanish Government Carlos III Health Institute Valencia Spain; ^5^ Department of Mental Health, Sanitary Research Institute of Valencia (INCLIVA) Hospital Clínico of Valencia Valencia Spain

**Keywords:** adherence, app, e‐health, psychosis, smartphone

## Abstract

**Aim:**

Despite the potential benefits of e‐health interventions for patients with psychosis, the integration of these applications into the clinical workflow and analysis of their long‐term effects still face significant challenges. To address these issues, we developed the ReMindCare app. This app aims to improve the treatment quality for patients with psychosis. We chose to study the app in real world and pragmatic manner to ensure results will be generalizable.

**Methods:**

This is a naturalistic empirical study of patients in a first episode of psychosis programme. The app was purpose‐designed based on two previous studies, and it offers the following assessments: (a) three daily questions regarding anxiety, sadness and irritability; and (b) 18 weekly questions about medication adherence, medication side effects, medication attitudes and prodromal symptoms. The app offers preset alerts, reminders and the ability for patients to reach out to their clinicians. Data captured by the app are linked to the electronic medical record of the patient. Patients will use the app as part of their ongoing care for a maximum period of 5 years, and assessments will occur at baseline and at the end of the first, second and fifth years of app use.

**Results:**

Recruitment started in October 2018 and is still ongoing.

**Conclusions:**

The ReMindCare app represents early real‐world use of digital mental health tools that offer direct integration into clinical care. High retention and compliance rates are expected, and this will in turn lead to improved quality of assessments and communication between patients and clinicians.

**Abbreviations**
App: application
e‐health: electronic health
EMR: electronic medical record
FEPP: first episode of psychosis program
SQ: satisfaction questionnaire

## 1 INTRODUCTION

Early intervention programs for first‐episode psychosis are effective evidence‐based interventions that foster recovery, prevent disability and reduce costs associated with illness in both the short and long term (Arango et al., 2017). However, like all clinical programs, they face implementation challenges. Specific challenges identified by the RAISE study in the United States include workforce development, community activation, fidelity and measurement of outcomes and patient involvement (Dixon, 2017). In Spain, which has a non‐contributory health system financed through taxation and supported by public funding, universal health coverage and free health care services result in a lack of professionals, tools and financial support to properly deliver these interventions (Arango et al., 2017). Thus, interventions that reduce the number of consultations and hospital admissions, enhance interview efficiency, increase the early detection of illness and enhance treatment efficacy are highly encouraged (Arango et al., 2018; MSCBS, 2019). Research on smartphone ownership among youth receiving early intervention services suggests that, like the rest of the population, they increasingly own these devices; a 2015 study suggested 81% ownership (Lal et al., 2015), and more recent research studies suggested 85% (Aref‐Adib et al., 2016) to 86% (Bonet et al., 2018) ownership. Furthermore, many studies have found high percentages of interest (70%‐80%) in e‐health interventions among these patients (Bonet et al., 2017; Firth et al., 2016; Gitlow et al., 2017), with no differences related to demographic or clinical characteristics (Berry, Lobban, Emsley, & Bucci, 2016; Bonet et al., 2018).

Thus, in this digital age, it is not surprising that those with psychosis are already turning to the internet and their smartphones for information about their illness, peer support and local treatment resources (Aref‐Adib et al., 2016; Torous & Keshavan, 2018), creating an opportunity for community engagement by using early intervention programs. Perhaps, the greatest potential for digital technology for early intervention programs is achieving fidelity and measurement of outcomes through automatically quantifying patients' treatment trajectory through real data, captured via surveys and sensors delivered via patients' phones. Numerous studies have shown the feasibility and acceptability of remotely monitoring location via smartphones and how such data can be used to predict risk and stratify patients with schizophrenia (Barnett et al., 2018; Bucci et al., 2018; Wang et al., 2016). By capturing and quantifying the lived experiences of those with psychosis, digital technology can help early intervention programs measure functional recovery metrics, such as employment, social support and medication adherence, in addition to subjective metrics.

However, despite the potential of digital technology to augment early intervention care, efforts to date have been largely composed of brief feasibility studies rather than actual clinical integration projects. Most interventions analysed have been implemented in periods ranging from 1 to 78 weeks, with the majority only implemented for 4 weeks or less (Berry et al., 2016; Bonet et al., 2017). A second limitation is the feasibility of translating these interventions into daily clinical practice, which has not been properly tested, as many studies were not designed to be implemented in the hospital or clinical workflow (Lauckner & Whitten, 2016; Zanaboni et al., 2018). Moreover, some studies suggested that, to achieve full integration into clinical practice, organizations also need to adapt their systems to these technologies; otherwise, success of the interventions would be harmed (Appelbaum & Wohl, 2000). A third limitation is the impact of digital health interventions on clinicians' workload and clinical efficiency remains understudied (Gitlow et al., 2017; Hoerbst & Schweitzer, 2015; Zanaboni et al., 2018). Finally, it is also important to consider patients' perspectives outside of clinical studies. Although the majority of studies found high levels of acceptance among patients in terms of participation in e‐health interventions (Berry et al., 2016; Bonet et al., 2018), some studies have indicated that excessive e‐health communications could be regarded as repetitive, intrusive or irritating (Kannisto, Adams, Koivunen, Katajisto, & Välimäki, 2015; Palmier‐Claus et al., 2013) or could increase worries about illness (Kannisto et al., 2015). Thus the pragmatic and real world nature of our study offers broad generalizable knowledge that considers not only how the app may impact care but also how it can be implemented into care.

The potential of digital mental health for early course psychosis stands in sharp juxtaposition to the limited real‐world clinical evidence for its impact, integration and acceptability among both patients and clinicians. To address these issues and improve the quality of early intervention programs in patients with a first episode of psychosis, we have developed an application (app) called “ReMindCare.” The ReMindCare app was created as a tool that could be integrated into standard psychiatric care and treatment, filling the gap between research and clinical practice.

## 2 METHODS

### 2.1 Study objectives

The main objective of this study is to address whether the introduction of the ReMindCare App into daily clinical practice improves the quality of treatment for patients in a first episode of psychosis programme (FEPP).

Specifically, the aims of the study are (1) to assess the effectiveness of the ReMindCare app, in terms of improvement of adherence to anti‐psychotic medication, early detection of relapses and improvement of communication with clinicians, vs treatment as usual in patients with a psychotic disorder; (2) to analyse the use of the ReMindCare app by the patients in terms of rates of adherence, compliance, alerts generated and the total time using the app; and (3) to assess the satisfaction of patients with using the app and the perceived usability of the ReMindCare app.

### 2.2 Study design

In the protocol, we describe the ReMindCare intervention as the first prospective naturalistic and empirical study of an app for FEPP. This app aims to improve the quality of the evaluation and treatment of patients. At the time of submitting this article, ReMindCare had been introduced as a clinical tool into daily psychiatric practice for more than a year. However, enrolment of patients continues, and the first analysis of the data will be conducted by March 2020.

### 2.3 Study setting

The ReMindCare app was systematically integrated into the daily FEPP workflow at the Public Clinical Hospital of Valencia (Spain), where it is currently being used. This FEPP is a free care service that aims to enhance the quality of the early care of outpatients with a first episode of psychosis, from the early phases of the illness through the first five critical years of treatment (Arango et al., 2017).

Given the pragmatic and naturalistic nature of this study, no remuneration or compensation is offered to patients participating in the programme or using the app. Rather, the app is offered as an additional and free service to the patients in treatment at study sites.

### 2.4 Participants

#### 2.4.1 Recruitment and enrolment

Every outpatient from the FEPP who meets the criteria for inclusion is considered a potential user of the ReMindCare app. Once patients enrol in the study, they are able to use the app for a maximum period of 5 years, which is the time when they would be discharged from the FEPP.

All patients interested in using the app must sign an informed consent form and must complete some baseline assessments before their inclusion in the study.

#### 2.4.2 Eligibility criteria

To be considered for this study, patients must be accepted into the FEPP at the Clinical Hospital of Valencia. Criteria for inclusion in the FEPP are:

Diagnosis of psychotic disorder following DSM‐5 (APA, 2013) criteria, interview conducted by a licensed clinician.
Less than 5 years of illness duration.
Residence associated with the hospital area of correspondence (Area 5 of Valencia).
Age between 17 and 65 years old
Ownership of a smartphone with an internet connection that allows the proper installation and functioning of the app.

Exclusion criteria include the following:

Inability to use and master a mobile device and the Internet.
Refusal to sign an informed consent form.
Spanish/English language fluency limiting ability to partake in clinical conversations or to understand the app questionnaires.

#### 2.4.3 Discontinuation and withdrawal

A patient's participation in the study will be discontinued if:

The patient provides an explicit notification of not wishing to continue using the app.
Lack of use the app for a period longer than 2 months after having been contacted by the research group over that period of inactivity.
Discharged from the FEPP or if their consent is revoked.
Given the naturalistic nature of this study, in case of discontinuation or withdrawal, patients will continue with the usual psychiatric treatment at the hospital.

### 2.5 Intervention

#### 2.5.1 Application development

The development process for the ReMindCare app can be divided into different phases, including review of the literature, a survey study, a design phase, a pilot study phase and a final version phase.

We first conducted a systematic review of previous publications on apps for psychosis (Bonet et al., 2017). Although the results of this review suggested that apps are feasible and well accepted by patients with psychosis, and apps can capture symptoms with good correlation to traditional metrics, a lack of clinical integration was notable across nearly all studies. Thus, to better understand how to design an app optimized for clinical integration, we next conducted a survey study of patient interests and preferences. We designed a survey based on previous publications (Borzekowski et al., 2009; Gay, Torous, Joseph, Pandya, & Duckworth, 2016; INE, 2016; Miller, Stewart, Schrimsher, Peeples, & Buckley, 2015; NAMI, 2014; Robotham, Satkunanathan, Doughty, & Wykes, 2016; Trefflich, Kalckreuth, Mergl, & Rummel‐Kluge, 2015) and administered it to a sample of 113 patients with psychosis. We aimed to evaluate the actual feasibility of e‐health interventions in a potential sample of patients with psychosis in our target population and to evaluate the interest of these patients in e‐health interventions (Bonet et al., 2018). The results highlighted that apps must offer improved communication with clinicians.

Next, we codesigned an e‐health app called ReMindCare with a team of clinicians, patients and developers. Our main objective of this process was to ensure the usability of the app. In this regard, both the patients and prior work in this space led us to focus on displaying information in the app graphically to easily allow a quick overview of the results and analysis of relevant information. We used a set of information technologies that simplified the development and communication between different parts of the platform. This included utilizing free and open source software tools such as the MongoDB database (MongoDB, GNU AGPL v3.0) to ensure flexibility and scalability, a Node.js server (Node.js, MIT Licence) to power the app, Docker containers (Docker, Apache Licence 2.0.) to protect privacy and frameworks such as Meteor and Bootstrap to build responsive user interfaces. This development of the app was conducted in a test environment outside the hospital network. Early efforts in this phase were focused on proper functioning of the database, the website and the mobile application.

With a functional first version of the ReMindCare app, we next focused on the challenges related to integration of the app into the hospital system. First, we had to study the current digital infrastructure of the hospital and adapt the platform to achieve its integration into the system while ensuring the performance of the app remained as designed, and patient data privacy was maintained. User authentication around the identification of both physicians and patients was a barrier that we faced. This identification was carried out through the Lightweight Directory Access Protocol (LDAP), which allows access to an organized and distributed directory service for an information search. The identification of patients and the report uploading was realized using Health Level Seven (HL7), which is an international set of standards to facilitate the electronic exchange of clinical information. This development process and installation of the app took approximately 2 years, with the most time spent on the clinical integration of the app into the hospital electronic system.

Although the process of integration was underway, we conducted a pilot study to test the validity and usability of the platform. This pilot trial involved four patients with psychosis for a period of 3 months, during which no negative effects associated with app use were found. The rates of compliance to surveys within the app were between 90% and 97%. Based on user feedback, we conducted further modifications to ensure the accurate functioning of the app in regards clinicians access to the app website, privacy of data registration and technical and electronic adjustments to achieve the automatic synchronization of ReMindCare with Android updates to ensure the appropriate performance of the app among different Android versions and smartphones.

#### 2.5.2 ReMindCare app

**FIGURE 1:**
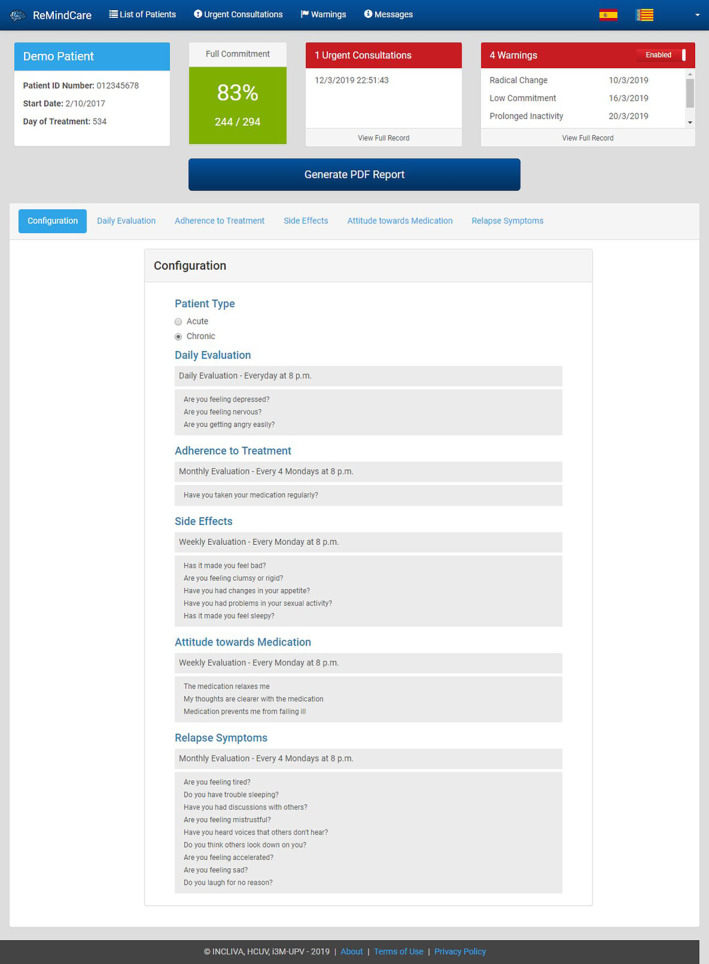
Screen shot of the ReMindCare dashboard (App for patients). Example of daily evaluation questions

ReMindCare is a free and user‐friendly app that conducts daily evaluations of the health status of patients with psychosis by offering quick questionnaires. Two types of questionnaires are presented (Figure 1):

- *Daily questionnaires*: Three daily questions that assess levels of anxiety, sadness and irritability.
- *Weekly questionnaires*: Eighteen weekly questions aimed to assess adherence to medication (1), the presence of side‐effects to anti‐psychotic medication intake (5), the attitude towards medication intake (3) and the presence of prodromal psychosis symptoms (9).

To use this clinical tool, clinicians enrol their patients via a private portal. Once the patients are registered into the portal, they can download the app, which is available on “Google Play,” log into the app and begin to use it. Patients are instructed on how to download and log into the app, and they are informed regarding how their app data are used.

**FIGURE 2:**
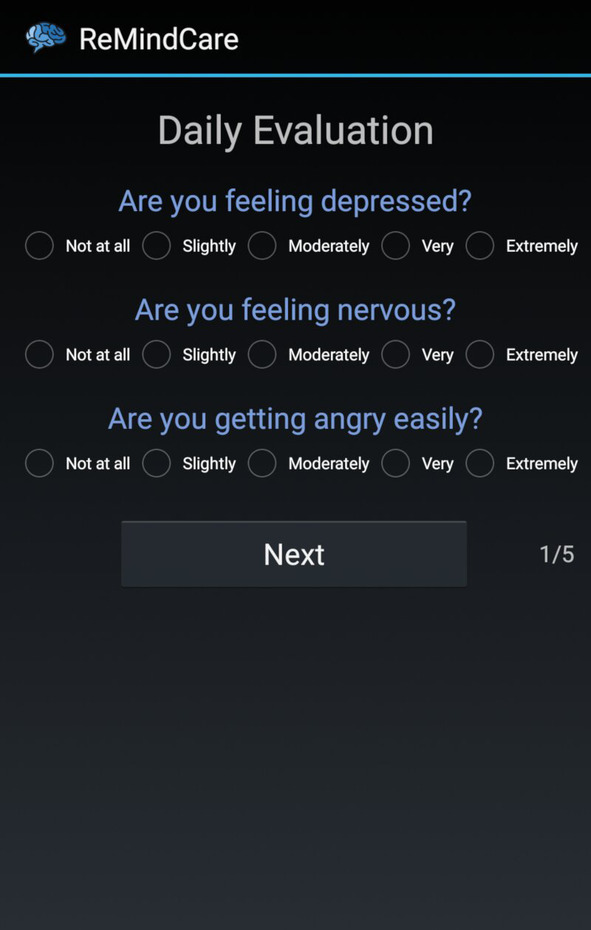
Screen shot of the ReMindCare dashboard (Website for clinicians). List of questions presented to patients

**FIGURE 3:**
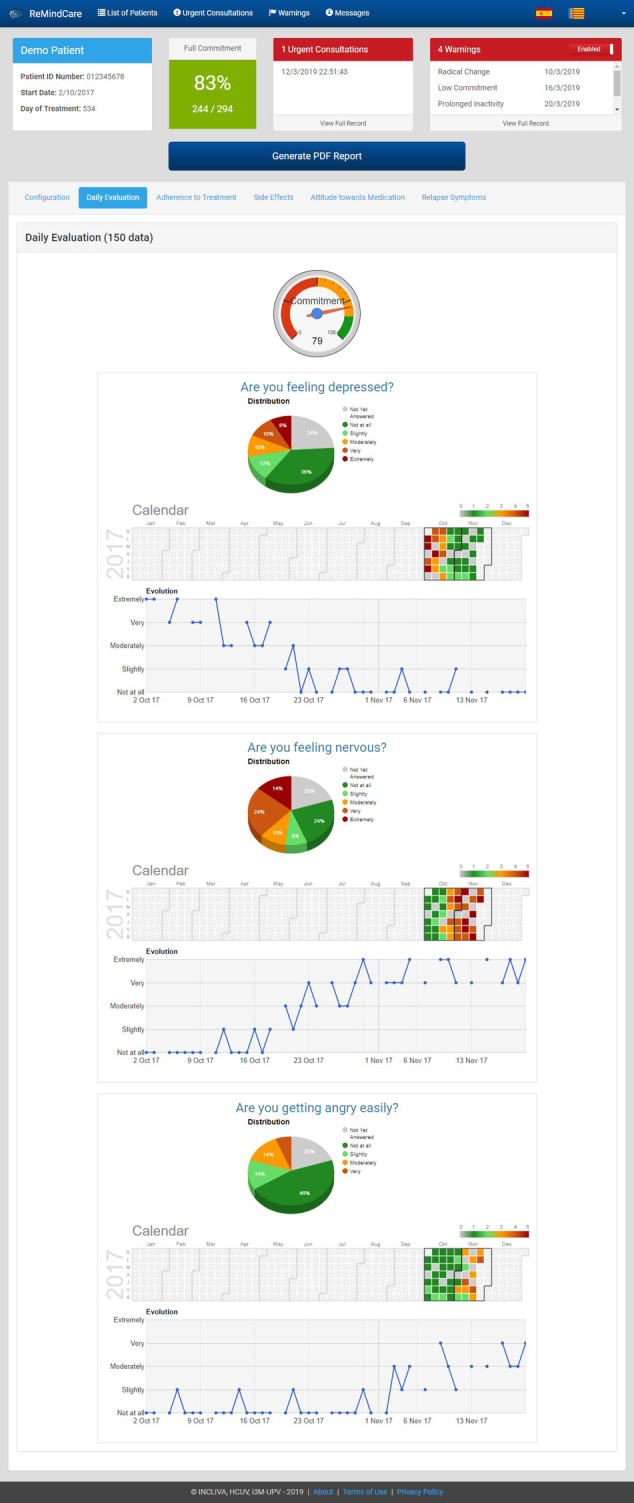
Screen shot of the ReMindCare Dashboard (for clinicians). Example of daily evaluation graphics

The information gathered by the app is accessible to clinicians on the portal, and it is exclusively used to orient clinicians for upcoming visits with patients, to help both patients and clinicians have a shared vision of the status of the patient and to discuss and establish together the therapeutic approaches. In addition, as displayed in Figures 2 and 3, on this portal, clinicians can not only visualize the information but also generate a PDF report that summarizes this information. These reports can be uploaded to the electronic medical record (EMR) of the patient because we enabled a function in the electronic hospital system that allows the inclusion of the ReMindCare app reports as another type of clinical report (eg, similar to results from a blood pressure test or glucose test). Moreover, if patients do not respond to notifications or abrupt variation in their answers occurs, the system automatically generates “alarms or warnings.” These alarms notify the clinician by e‐mail and are also displayed in the profile of the patient on the website. Moreover, patients are able to contact their clinician using an “Urgent Consultation” tab displayed on the app if they detect a significant worsening in their health status. By clicking this button, clinicians receive a notification e‐mail and have to contact the patient within a maximum period of 48 hours. Further information about the use of the app and the webpage is available in the user manual included in Appendix A.

### 2.6 Data collection and measures

An overview of all included measures and assessment points is included in Table 1.

**TABLE 1:** Study measures and assessment times

Timepoint	ReMindCare intervention					
	Baseline	Intervention			Follow‐up	
	*t*_0_	*t*_1_	*t*_2_	*t*_3_	*t*_4_	*t*_5‐8_
Enrolment						
Eligibility screen	X					
Informed consent	X					
Assessments						
Baseline assessment						
Clinical Global Impression Scale (CGI) (Busner & Targum, 2007)	X				X	X
Global Assessment of Functioning (GAF) (Endicott, Spitzer, Fleiss, & Cohen, 1976)	X				X	X
Positive and Negative Syndrome Scale (PANSS) (Peralta & Cuesta, 1994)	X				X	X
Premorbid Adjustment Scale (PAS) (Cannon‐Spoor, Potkin, & Wyatt, 1982)	X				X	X
Drug Attitude Inventory (DAI‐10) (Hogan, Awad, & Eastwood, 1983)	X				X	X
Beck Cognitive Insight Scale (BCIS) (Beck, Baruch, Balter, Steer, & Warman, 2004)	X				X	X
Sociodemographic information (Age, sex, ethnicity, marital status, level of education, living situation and employment status)	X					
Clinical information (Diagnose, years of illness, pharmacological treatment, suicidal attempts, history of illness)	X		X		X	X
Outcome measures						
Simplified Medication Adherence Questionnaire (SMAQ) (Morisky, Green, & Levine, 1986)	X		X		X	X
Number of relapses	X		X		X	X
Number of visits to urgent care units at the hospital	X		X		X	X
Number of hospital admissions	X		X		X	X
ReMindCare measures						
Answers to questionnaires		X	X		X	X
Quantity of “urgent consultation” requests		X	X		X	X
Quantity of alarms generated		X	X		X	X
Satisfaction Questionnaire^†^				X		X

Note: *t*_1_ = introduction of the ReMindCare app into clinical practice; *t*_2_ = first‐year preliminary data assessment; *t*_3_ = focus group, first year; *t*_4_ = second‐year follow‐up; *t*_5‐8_ = fifth‐year follow‐up; Satisfaction questionnaire^†^ = App feedback questionnaire made for the purpose of this research.

Baseline surveys, including clinical and sociodemographic information and clinical standardized questionnaires, were administered before the enrolment of the patient in the study. As shown in Table 1, data regarding the main outcomes, such as adherence to medication, number of relapses, number of visits to the hospital urgent care units and number of hospital admissions, will be collected and analysed at the end of the first, second and fifth years of the intervention. Moreover, information generated for patients through the use of the app will also be analysed at the same timepoints. Finally, to analyse the feedback of patients regarding the use of the app, we plan to conduct focus groups at the end of the first year of the intervention, and we have also designed a “satisfaction questionnaire” (SQ) that patients will complete at the end of the first year of the intervention or before discontinuing the use of the app (if discontinuation occurs before the first year of app usage). This SQ was made for the purpose of this research study and is based on previous satisfaction and usability questionnaires, such as the user version of the Mobile Application Rating Scale (uMARS) (Stoyanov, Hides, Kavanagh, & Wilson, 2016), the System Usability Scale (SUS) (Brooke, 1996), EnLight: a tool for mobile and web‐based eHealth interventions (Baumel, Faber, Mathur, Kane, & Muench, 2017) and the App Quality Evaluation (AQEL) (DiFilippo, Huang, & Chapman‐Novakofski, 2017). This questionnaire is displayed in Appendix B.

### 2.7 Planned data analysis

Descriptive analyses of sociodemographic and clinical variables will be conducted. A multivariate data analysis will be carried out to explore relationships between sociodemographic and clinical variables and adherence to treatment and ReMindCare measures. An ANOVA model of repeated measures will be used for the main research outcomes. All analyses will be conducted with an alpha set at *P* < 0.05.

These preliminary quantitative data analyses and the preliminary results regarding patient compliance, retention rate and perceived satisfaction with the use of the ReMindCare app are expected by the end of the first year of using the app (March 2020). At that time, qualitative analyses of data are also planned.

Subsequent analysis of the data will be conducted at the end of the second and fifth years of use of the app.

### 2.8 Ethics, data privacy and participant safety

The ReMindCare app project has received full approval from the Research Ethics Committee of the faculty of Medicine at the University of Valencia and from the Research Ethics Committee of the Sanitary Research Institute (INCLIVA) of the Clinical Hospital of Valencia, Spain.

To protect the data sent by patients, communications to the platform are encrypted with a transport layer security certificate from the Generalitat Valenciana and are sent through the HTTPS (Hypertext Transfer Protocol Secure) protocol. This process ensures that all transmitted data are completely private and without a chance of being manipulated. Moreover, the hospital infrastructure is protected through a reverse proxy, which enhances security by establishing a single access point to it and hiding all inner infrastructures. Moreover, the integration of the app into the hospital systems is subjected to the LOPD‐GDD (Organic Law 3/2018: protection of personal data and digital rights guarantee, December 5th), the Spanish organic law adaptation of the GDPR (General Data Protection Regulation).

## 3 RESULTS

The ReMindCare app was systematically introduced into clinical practice in October 2018, and 57 patients have been enrolled in the study since then. Updates of the app and improvements in its functioning will be conducted as requested by feedback obtained from patients and clinicians and in accordance with Android and iOS developments.

## 4 DISCUSSION

ReMindCare is an e‐health intervention aimed at improving the quality of the current programme for early treatment of patients with psychosis. Although there are an increasing amount of apps being studied for early course psychosis (Camacho, Levin, & Torous, 2019), to our knowledge, this is the first pragmatic and prospective integration of an app into real world clinical care. The study is thus designed to generate data beyond just how the app may improve care but also how it can be implemented into clinical care, leading to the ability to augment quality existing health services (Bonet et al., 2017).

### 4.1 Anticipated results

On the basis of previous studies (Bonet et al., 2017; Firth et al., 2016; Gitlow et al., 2017) that confirmed the interest of patients with psychosis in e‐health interventions and based on the high interest in owning an e‐health app that we found in our survey study (Bonet et al., 2018), we expect an acceptable retention rate (beyond 70%).

Moreover, based on previous studies (Ben‐Zeev et al., 2014; Ben‐Zeev, Kaiser, & Krzos, 2014; Brenner & Ben‐Zeev, 2014; Kimhy, Vakhrusheva, Liu, & Wang, 2014; Macias et al., 2015) that found rates of response to the communications of e‐health apps to be higher than 70%, we also expect moderate compliance with the app's communications (beyond 70%).

Furthermore, as previous studies have stated that electronic assessments are valid and reliable measures (Brenner & Ben‐Zeev, 2014; Kimhy et al., 2014; Palmier‐Claus et al., 2013; Spaniel et al., 2008), and based on preliminary perceptions obtained from the first 7 months of the current intervention, we expect that the use of the ReMindCare app would produce the following differences in the treatment of psychotic disorders:

- *Improved quality of treatment delivery for clinicians*: Graphics of daily and weekly questionnaires allow clinicians to rapidly assess the status of the patient and to specifically orient their interactions with the patient to problematic areas. An improvement in the quality of evaluation is also expected. Perceptions of the clinicians regarding these issues will be qualitatively assessed in focus groups.
- *Improved insight about the illness and mental health status for patients*: Sharing with patients their responses to the app is expected to increase accuracy of information and to decrease bias in this process. Moreover, it is expected that, by discussing their app responses with the patients, insight about their illness and their health status will increase. These perceptions will be mainly assessed by the “satisfaction questionnaire” (SQ), and they will also be assessed qualitatively in focus groups. Moreover, changes in insight between baseline and 1 year of intervention will be assessed by using the BCIS (Beck et al., 2004).
- *Improved quality of communication between patient and clinician*: It is expected that patients will feel more understood as a result of improvements in evaluation and quality of their interactions with the clinician, which could lead to an enhancement of the overall quality of communication and alliance between patient and clinician. Again, this will be assessed by the SQ and the focus groups.
- *Improved adherence to treatment for patients*: As a result of improvements in insight about the illness and improvements in alliance with clinicians, an enhancement of adherence to treatment is also anticipated. Significant differences are expected in the SMAQ (Hogan et al., 1983) scores of patients between baseline and 1 year of intervention. Moreover, the number of relapses, treatment dropouts and rates of compliance and adherence to ReMindCare tests will also be analysed in this regard. It is expected that the more patients use ReMindCare (high rates of engagement and compliance), the fewer treatment dropouts and relapses there will be.
- *Improved early relapse detection and hospital admission reduction*: Again, higher rates of engagement with ReMindCare are expected to produce lower hospital admissions, early detection of relapses and fewer visits to urgent care units. Moreover, rates of relapses, changes in medication and visits to urgent care units will be compared among patients who used the “urgent consultation” function and those who do not. We expect that using this function will improve the quality of early detection.
- *Improved quality of communication between health care providers and improved quality of treatment decisions*: Due to general access of health care professionals to ReMindCare reports on the EMR, we expect improved communication between health professionals and, because of this, improved quality of treatment. Perceptions of clinicians on this issue will be qualitatively assessed in focus groups.

### 4.2 Strengths and limitations

The main strength of our approach is the simplicity of the app and the direct integration of ReMindCare into daily clinical practice. As stated before, one of the most important factors regarding the sustainability of e‐health interventions relies upon good integration into the workflow of health systems (Abbott, Foster, Marin Hde, & Dykes, 2014; Appelbaum & Wohl, 2000; Cresswell & Sheikh, 2013; Granja, Janssen, & Johansen, 2018). The ReMindCare app was designed with the main objective of being useful not only for patients but also for health providers. In this regard, the user‐friendly design of the app, its integration into the public hospital workflow and the free access to the app for patients are major strengths that aim to ensure the use of the app by both patients and clinicians and to improve, as a result, the quality of treatment that patients receive and the quality of health services that clinicians provide.

Another main strength of our study is that the development of the app was based on two previous studies (Bonet et al., 2017, 2018), which allowed us to be confident about the theoretical framework of the intervention and to truly address the necessities of the patients. As we found in our study (Bonet et al., 2018), patients claimed improvements in communication with clinicians, which was one of our main objectives when we designed the app.

The graphical display of information can be regarded as another major strength. The integrated graphics provide a quick overview of the health status of the patient, which is extremely useful in the context of a busy public health system (Arango et al., 2018). Specifically, these graphics can help clinicians detect side‐effects of medication that are not usually commented on by patients, such as sexual dysfunctions. This is relevant, as medication side‐effects are one of the most important factors affecting medication dropouts (García et al., 2016).

Finally, the ReMindCare app has been developed in three languages, Spanish, English and Catalan, which allows the use of the app in different countries and autonomous communities.

However, some limitations must be taken into consideration. First, because of the characteristics of this real‐world intervention, our study is not randomized or controlled. However, we plan to compare the main outcomes of the use of the app between users and age‐matched controls, although the groups may differ in some characteristics. Another limitation is that ReMindCare has only been designed for Android systems, although an iOS version of the app is being designed.

To our knowledge, this is one of the earliest e‐health interventions for patients with psychosis implemented as a standard care tool integrated into clinical practice in the public hospital workflow. Real‐time health information is being collected and used to work together with patients to improve the quality of real‐world health care delivery.

## Supporting information

Appendix A. User manual

Appendix B. Satisfaction questionnaire

## Data Availability

The data that support the findings of this study are openly available in [repository name e.g “figshare”] at http://doi.org/[doi], reference number [reference number]
