# Supplementary material for: ReMindCare, an app for daily clinical practice in patients with first episode psychosis: A pragmatic real‐world study protocol
Source: Early Interv Psychiatry. 2020 Apr 6;15(1):183–92. doi: 10.1111/eip.12960 (PMC7891598; doi:10.1111/eip.12960)
Supplement: Supplementary file 1 — Appendix A. User manual [file EIP-15-183-s001.pdf]

ReMindCare

REMINDCARE APP

USER MANUAL

Clinician Version

ReMindCare App User Manual:

An app designed by the Unit of Psychiatry at the Clinical Hospital of Valencia in liaison with the Polytechnic University of Valencia.

The ReMindCare app is aimed to improve assessment of the clinical health status of patients with a psychotic disorder diagnosis and communication between patient and clinician.

GENERALITAT VALENCIANA
CONSELLERIA DE SANITAT UNIVERSAL I SALUT PÚBLICA
DEPARTAMENT DE SALUT DE VALÈNCIA CLÍNIC-LA MALVA-ROSA
INCLIVA Instituto de Investigación Sanitaria
UNIVERSITAT POLITÈCNICA DE VALÈNCIA
i3M Instituto de Instrumentación para Imagen Molecular

INDEX

0. APP DESCRIPTION 1

1. FUNCTIONING: HOW DOES IT WORK? 1

0. GENERAL FUNCTIONING ………………………………………..……… 1
1. REGISTRATION OF THE CLINICIAN INTO THE SYSTEM ……….. 2
2. ACCESS TO CLINICIAN´S PROFILE ….……………………………… 2

2.1 **List of patients**
2.2 **Urgent consultations**
2.3 **Alerts**
2.4 **Messages**

3. REGISTRATION OF THE PATIENT INTO THE SYSTEM …….…... 6
4. PATIENT DATA DISPLAY ……………………………………..………. 7

4.1. **Activity summary boards:**

1. *Patient identification board*
2. *Total compliance board*
3. *Urgent consultation notifications board*
4. *Alert notification board*

4.2. **Button to generate pdf resume-report.**

4.3. **Data tabs:**

a. *Set up*
b. *Daily evaluation*
c. *Adherence*
d. *Side effects*
e. *Attitude towards medication.*
f. *Prodromal symptoms of relapse.*

5. APP INSTALLATION INTO PATIENTS SMARTPHONE 15

6. APP FUNCTIONING 15

6.1. Notification presentation

6.2. Response time frame

6.3. Questionnaires:

a. *Mood status daily evaluation*
b. *Adherence to medication weekly evaluation*
c. *Medication side effects weekly evaluation.*
d. *Attitude towards medication weekly evaluation.*
e. *Prodromal symptoms of relapse weekly evaluation*

6.4. Alerts system:

a. *Alerts automatically generated by the system:*
    a. Prolonged inactivity.
    b. Low Compliance
    c. Abrupt change.
b. *Alerts deliberately generated by the patient.*

APPENDIX I. REPORT EXAMPLE... 25

ReMindCare is an e-Health app that gathers information about the clinical health status of patients with a Psychotic Disorder diagnosis through brief daily and weekly assessments.

This information is displayed in a restricted access website, where clinicians can visualize data from patients as well as download pdf reports of the main data collected by the app. These reports can be attached to the electronic clinical report of the patient at the hospital database and are accessible for consultation by every clinician involved in the treatment of the patient.

Furthermore, ReMindCare produces different alarms that notify clinicians about variations in patient health status or the cessation of using the app. Moreover, patients can also deliberately generate an urgent consultation alarm.

The process of design and implementation of the app can be divided into different phases:

First, we conducted a systematic review of previous publications (Bonet et al. 2017). Our aim was to assess trends in e-Health app development as well as their potential benefits in the treatment of patients with psychosis. As a result of this study, we reached the following conclusions: e-Health interventions are feasible and well accepted by patients, and they can improve treatment of the illness. However, most e-Health interventions were not implemented in hospital daily workflow or have not been implemented for more than 2 years; consequently, there was a lack of long-term analysis.

Second, our aim was to evaluate the actual feasibility of e-Health interventions in a potential sample of patients with psychosis in our target intervention population; we also aimed to evaluate the interest of these patients in e-Health interventions and in different e-Health services presented (Bonet et al. 2018). As a result of this second study, we determined the viability of e-Health interventions in our study sample and the interest of these patients in e-Health apps. Special rates of interest were registered in app services related to improvement of communication with clinicians.

Third, we designed the app and named it ReMindCare. Our main objective when designing this app was to guarantee its usability and improvement of communication between patient and clinician. To achieve these objectives, a set of technologies that ease the development and communication between different parts of the platform has been used. First, patient data are gathered into a MongoDB database (MongoDB, GNU AGPL v3.0), which ensures flexibility and scalability. Second, the website works on a Node.js server (Node.js, MIT License), isolated in a Docker container (Docker, Apache License 2.0.), and it has been developed with frameworks such as Meteor and Bootstrap. Mobile application was implemented with Android and iOS

native code to increase its performance. All of these products and technologies, except iOS, are free and have open-source licenses.

Finally, we conducted a pilot study to test its validity and usability. A pilot trial involving 4 patients was conducted for a period of 3 months, and no negative effects associated with the use of the app were found. The rates of compliance to test displayed for the app were between 90% and 97%. However, it must be noted that one patient abandoned the study 5 days after enrolment because he had a worsening in his medical status that required hospital admission.

As a result of this pilot test, we validated the usability of the app among patients. However, some modifications regarding clinician access to the app website, privacy of data registration and some technical and electronical adjustments are required.

Bonet L, Izquierdo C, Escartí MJ, Sancho JV, Arce D, Blanquer I et al. Utilización de tecnologías móviles en pacientes con psicosis: una revisión sistemática. Rev Psiquiatr Salud Ment 2017 Jul-Sept; 10(3): 168-78. PMI: 28258835

Bonet L, Llácer B, Hernandez-Viadel M, Arce D, Blanquer I, Cañete C et al. Differences in the Use and Opinions About New eHealth Technologies Among Patients With Psychosis: Structured Questionnaire. JMIR Ment Health. 2018 Jul;5(3):e51. PMID: 30045835

0. APP DESCRIPTION:

ReMindCare is an app that conducts daily evaluations of the clinical status of patients with a psychotic disorder diagnosis. This study aimed to improve the evaluation of their health status and communication between patients and clinicians. This app was initially designed exclusively for patients from the First Episode of Psychosis Unit (FEPU) of the psychiatry unit at the Clinical Hospital of Valencia.

1. FUNCTIONING: HOW DOES IT WORK?

0. GENERAL FUNCTIONING:

To start using the service, clinicians must register in the ReMindCare application system. Subsequently, clinicians need to register each patient into the system. Once the patient has been registered into the system by the clinician and has downloaded the app into his/her smartphone, the patient will receive different notifications daily to answer questions aimed at assessing his/her health status.

Information gathered by the application based on these assessments, will be displayed on ReMindCare´s webpage and can be used to generate alerts if abrupt variations in patient responses are detected. Furthermore, inside the app, the patient can find a button labelled “Urgent Consultation” that he/she can click to inform his/her clinician about a potential worsening in his/her mental status.

Information gathered by the application about patients as well as information related to alerts generated by the system will be uniquely displayed in the profile of the clinician who has registered each patient.

GENERALITAT VALENCIANA CONSELLERIA DE SANITAT UNIVERSAL I SALUT PÚBLICA
DEPARTAMENT DE SALUT DE VALÈNCIA CLÍNIC-LA MALVA-ROSA
INCLIVA Instituto de Investigación Sanitaria
UNIVERSITAT POLITÈCNICA DE VALÈNCIA
i3M Instituto de Instrumentación para Imagen Molecular

1. REGISTRATION OF THE CLINICIAN INTO THE SYSTEM:

To register into the system, clinician needs to perform the following steps:

1. Access the ReMindCare website: https://158.42.105.14/.
2. Click the "Sign in" tab on the top right corner of the page.
3. Type in his/her e-mail address, password, given name and surname.
4. Click the "Register" button.

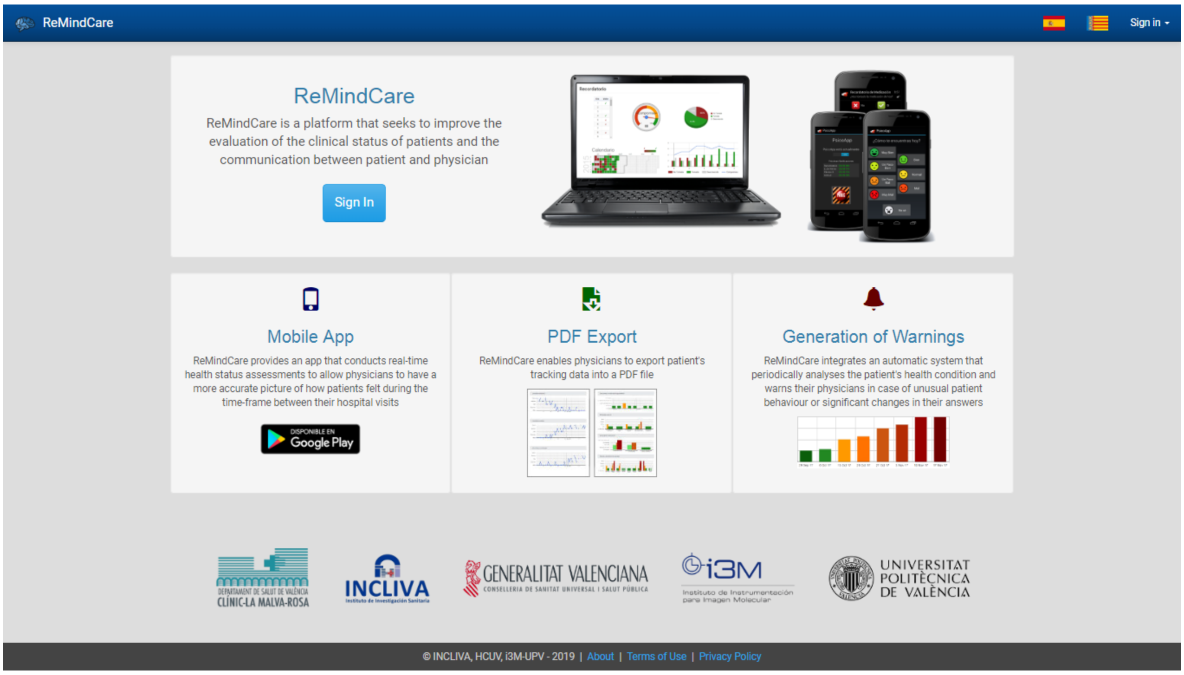

Screenshot of the front page of the ReMindCare website

2. ACCESS TO CLINICIAN PROFILE:

To access his/her profile, the clinician needs to perform the following steps:

1. Click the "sign in" tab on the top right corner of the page.
2. Type in his/her e-mail and password.
3. Click the "Sign in" button.

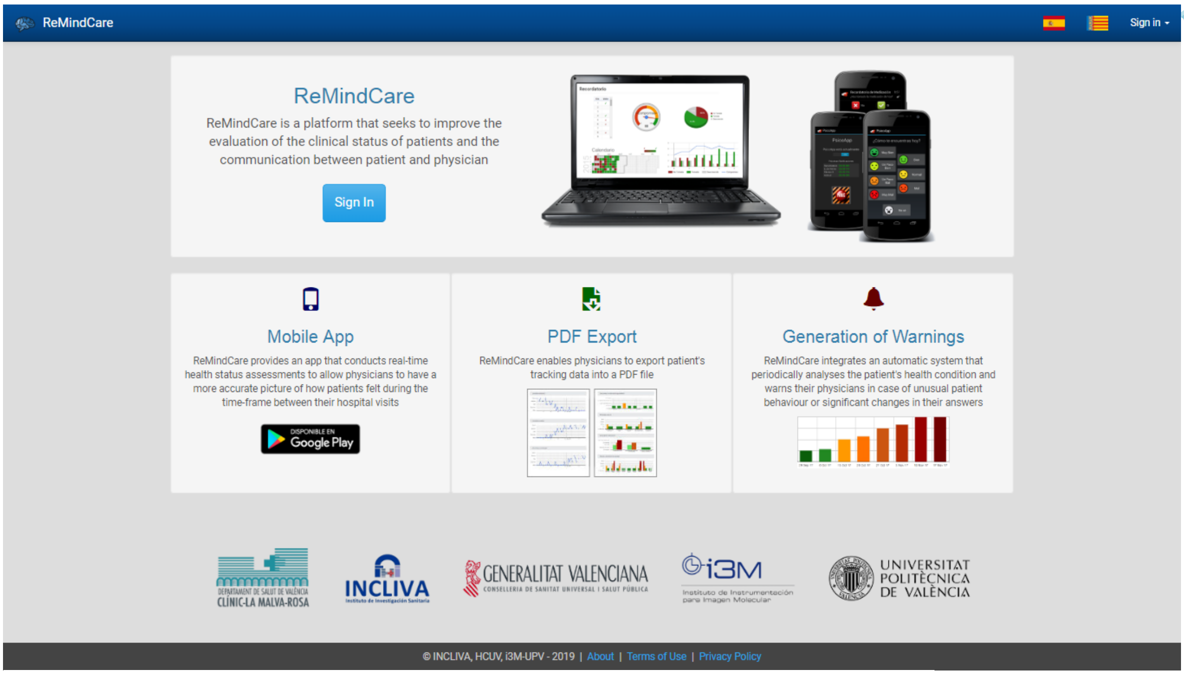

Screenshot of the front page of the ReMindCare website

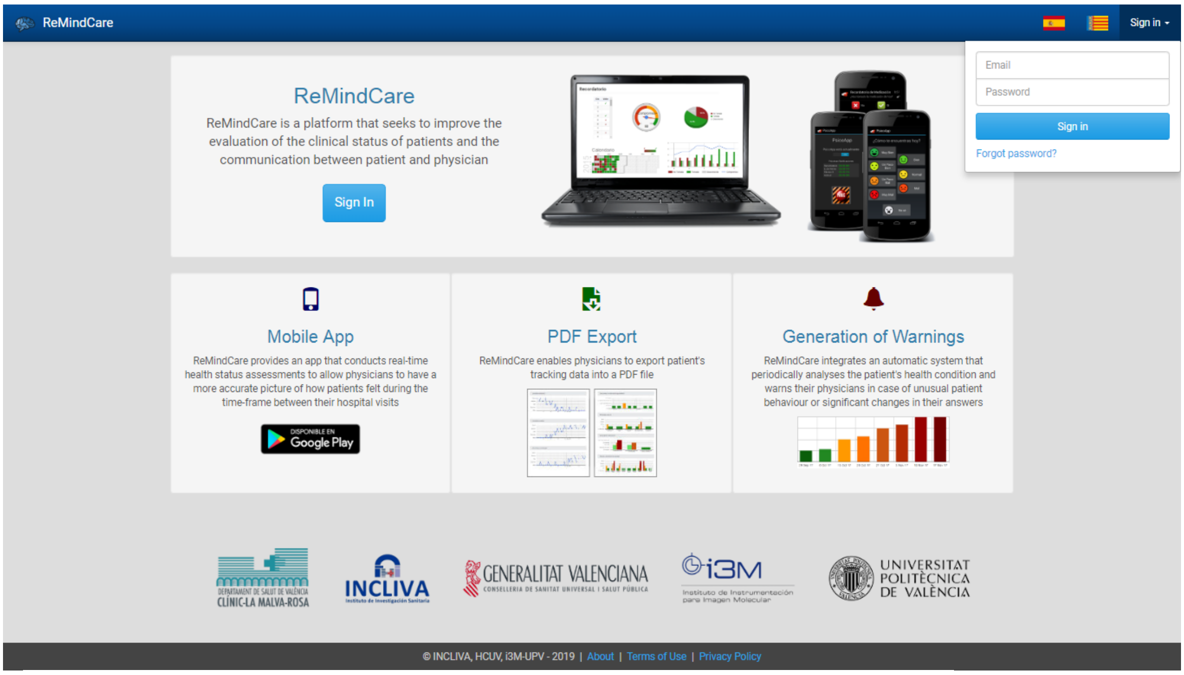

Screenshot of the front page of the ReMindCare website

Once the clinician has logged into the system, he/she will have access to his/her profile where four buttons are displayed:

2.1 List of patients:

Displays the list of logged patients and a summary of their main characteristics:

a. Patient identification code (SIP number).
b. Date of registration.
c. Days of treatment or of using the app.
d. Compliance or percentage of global response to app notifications.
e. Number of urgent consultation notifications generated for the patient.
f. Number of alert notifications generated for the app.
g. App status (Active or linking pending).

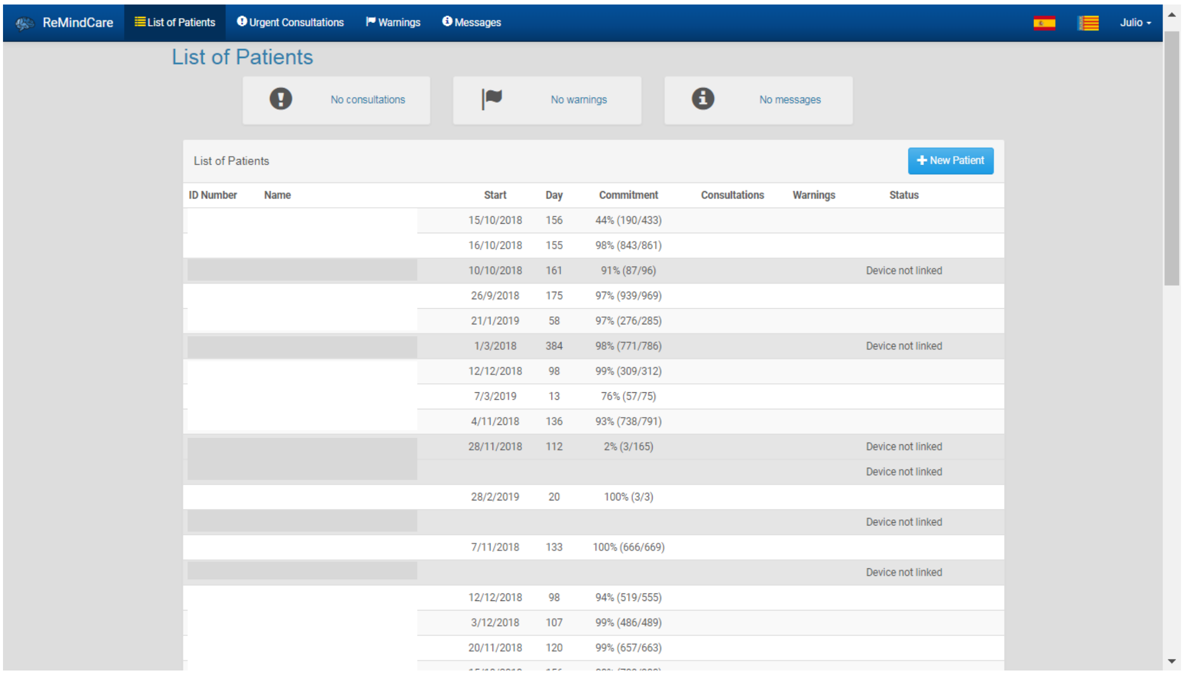

ID Number	Name	Start	Day	Commitment	Consultations	Warnings	Status
		15/10/2018	156	44% (190/433)			
		16/10/2018	155	98% (843/861)			
		10/10/2018	161	91% (87/96)			Device not linked
		26/9/2018	175	97% (939/969)			
		21/1/2019	58	97% (276/285)			
		1/3/2018	384	98% (771/786)			Device not linked
		12/12/2018	98	99% (309/312)			
		7/3/2019	13	76% (57/75)			
		4/11/2018	136	93% (738/791)			
		28/11/2018	112	2% (3/165)			Device not linked
							Device not linked
		28/2/2019	20	100% (3/3)			
							Device not linked
		7/11/2018	133	100% (666/669)			
							Device not linked
		12/12/2018	98	94% (519/555)			
		3/12/2018	107	99% (486/489)			
		20/11/2018	120	99% (657/663)			

Screenshot of the list of patients from the ReMindCare website.

2.2 Urgent consultation:

This board allows access to information related to notifications that patients have generated deliberately. In this section, information about patients who have generated notifications and their characteristics (date and time) are displayed.

2.3 Warnings:

Allows access to information related to notifications that the system has automatically generated. This information can be expressed as follows:

a. Prolonged inactivity
b. Abrupt change
c. Low Compliance

2.4 Messages:

Allows the creation and access to messages that clinicians consider relevant to include.

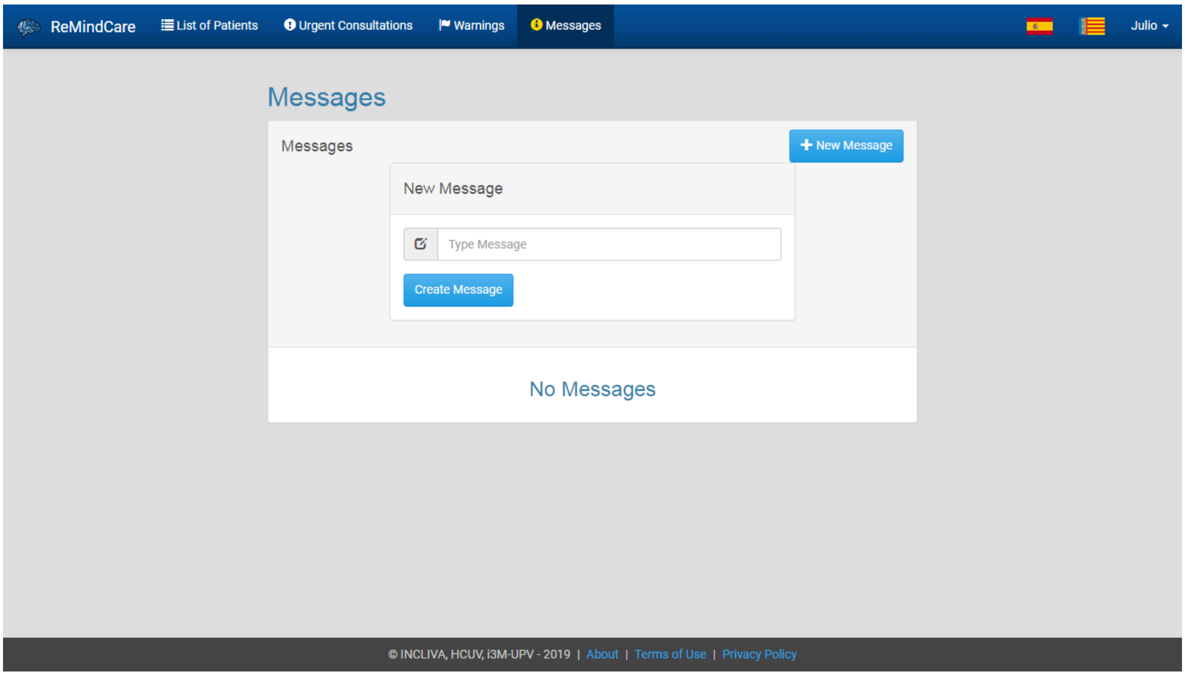

Screenshot of the message window on the website.

3. REGISTRATION OF THE PATIENT INTO THE SYSTEM:

To register the patient into the system, the clinician needs to type in the patient´s hospital identification number (SIP code) along with the patient's given name and surname according to the following steps:

1. Access the ReMindCare website: https://158.42.105.14/.
2. Access the clinician's personal profile (See Section 2. Access to clinician profile).
3. Click the "List of patients" tab, follow by another click on the "+New Patient" button.
4. Type in the patient´s hospital identification number (SIP code) along with his or her given name and surname.
5. Click the "Enroll patient" button.

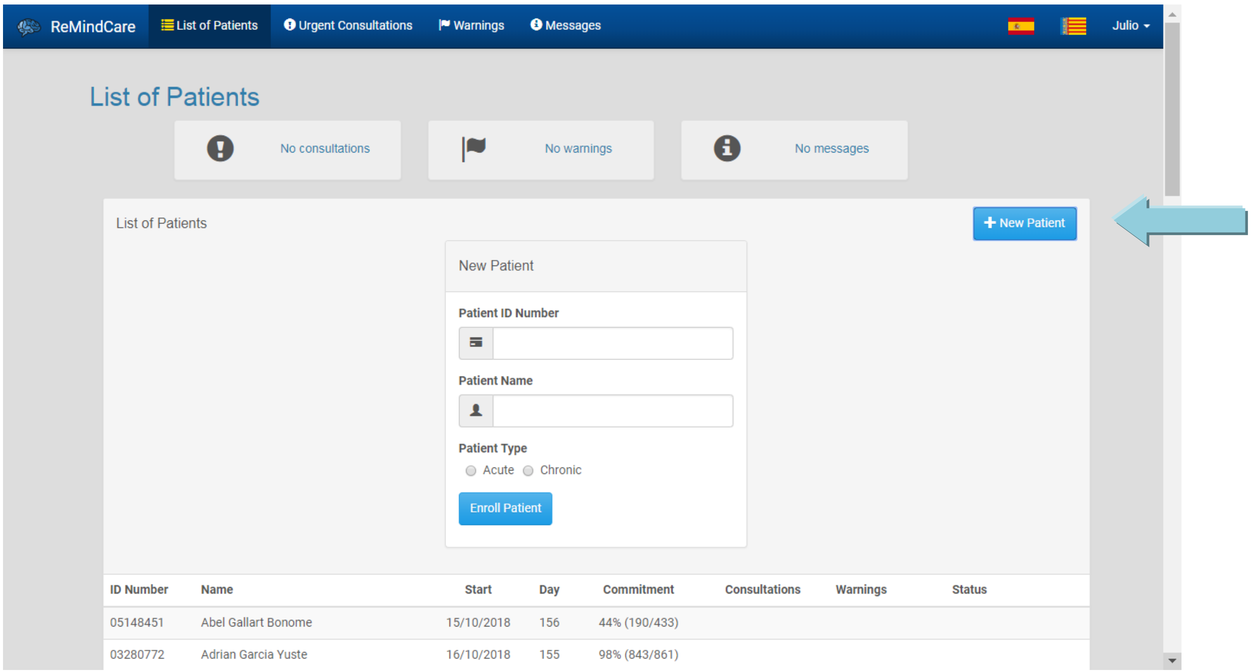

Screenshot of the patient registration tab on the ReMindCare website.

4. PATIENT DATA DISPLAY:

Once the patient has been registered and has started making use of the app, the clinician would be able to view data gathered by the app on this website. To view patient data, the clinician must:

1. Access his or her profile.
2. Click the name of the patient whose data are to be visualized.

By clicking on a specific patient, we can access his/her profile. In this profile, the following data gathered by the app are displayed:

4.1. Activity summary boards:

a. ***Patient identification board:*** Contains information related to the patient and his/her registration into the system:
   – Hospital identification number (SIP code)
   – Date of registration
   – Days of treatment or of using the app.

b. ***Full Commitment****:* Displays the total percentage and number of responses to app notifications that the patient has made.

c. ***Urgent consultation notification board:*** Displays the time and date of the urgent consultation alert generated by the patient.

d. ***Warning notification board:*** Displays the date and type of the alert generated for the system in relation to patient responses.

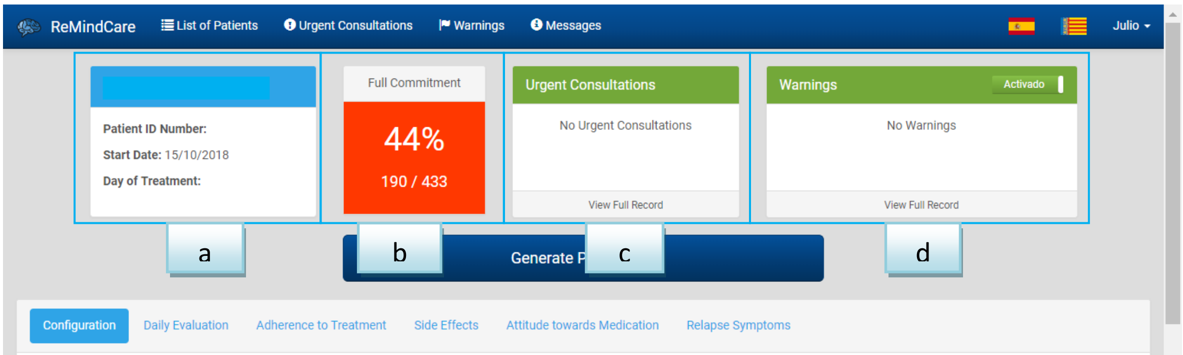

Screenshot of the summary boards page on the website.

4.2. Button to generate pdf summary-report:

Under activity summary boards, there is a button called “Generate PDF Report”. By clicking into this button, a pdf containing the main data collected by the app will be downloaded. A sample of this report is available at **APPENDIX I.**

Once the report has been downloaded, it can be attached to the electronic clinical history of the patient at his/her hospital of preference. It can be visualized just like any other clinical report.

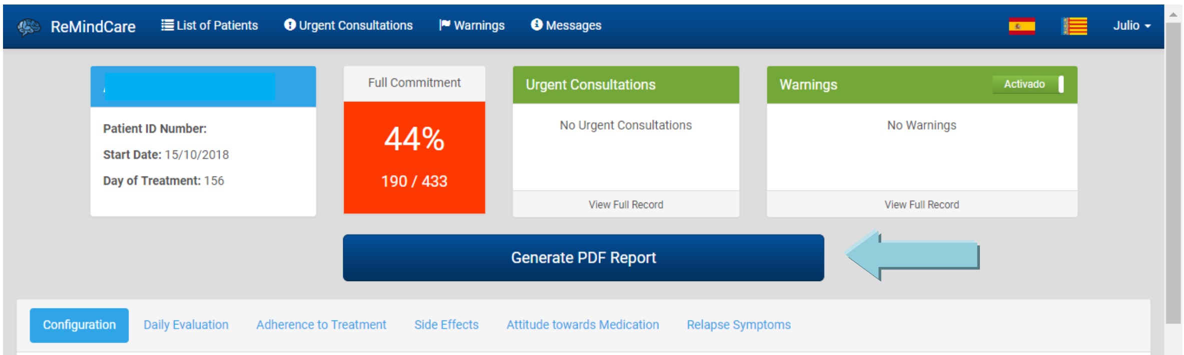

Screenshot showing the button used to generate a pdf summary report on the website.

4.3. Data tabs:

Composed of one setup tab and five tabs that allow access to data gathered by the app.

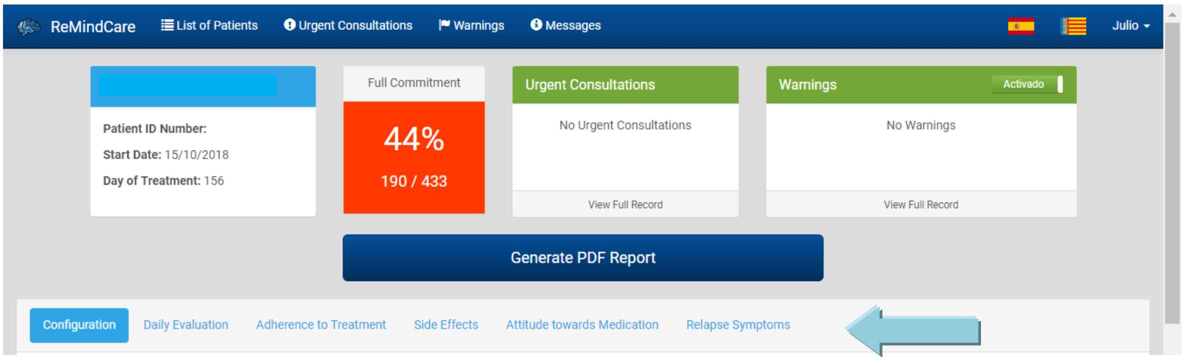

Screenshot of website tabs.

a. Configuration tab:

Allows modification of patient data.

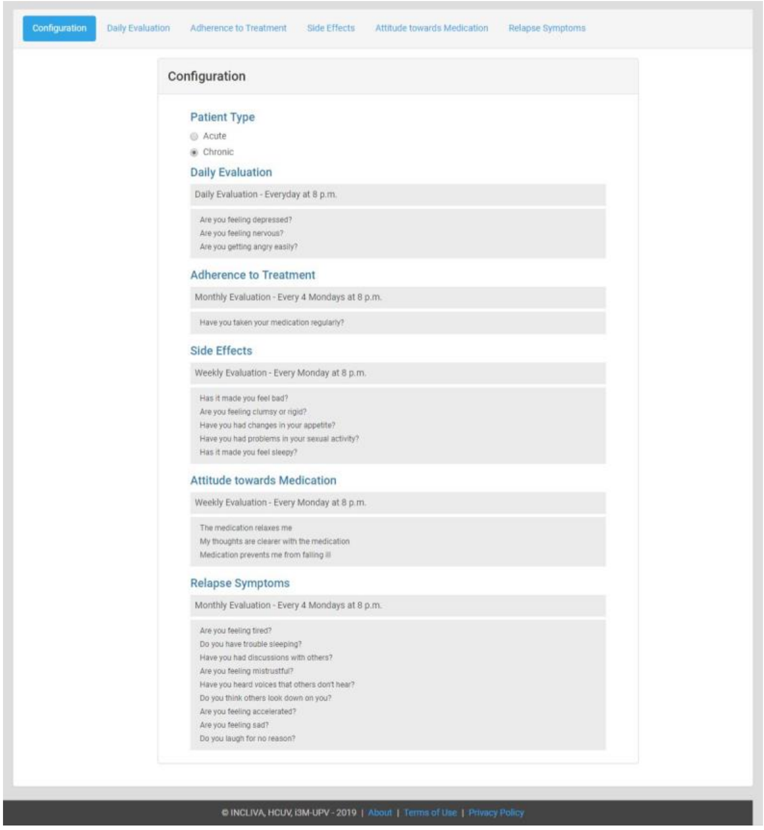

Screenshot of configuration tab from the website.

b. Daily evaluation tab:

Allows access to data gathered daily by the app.

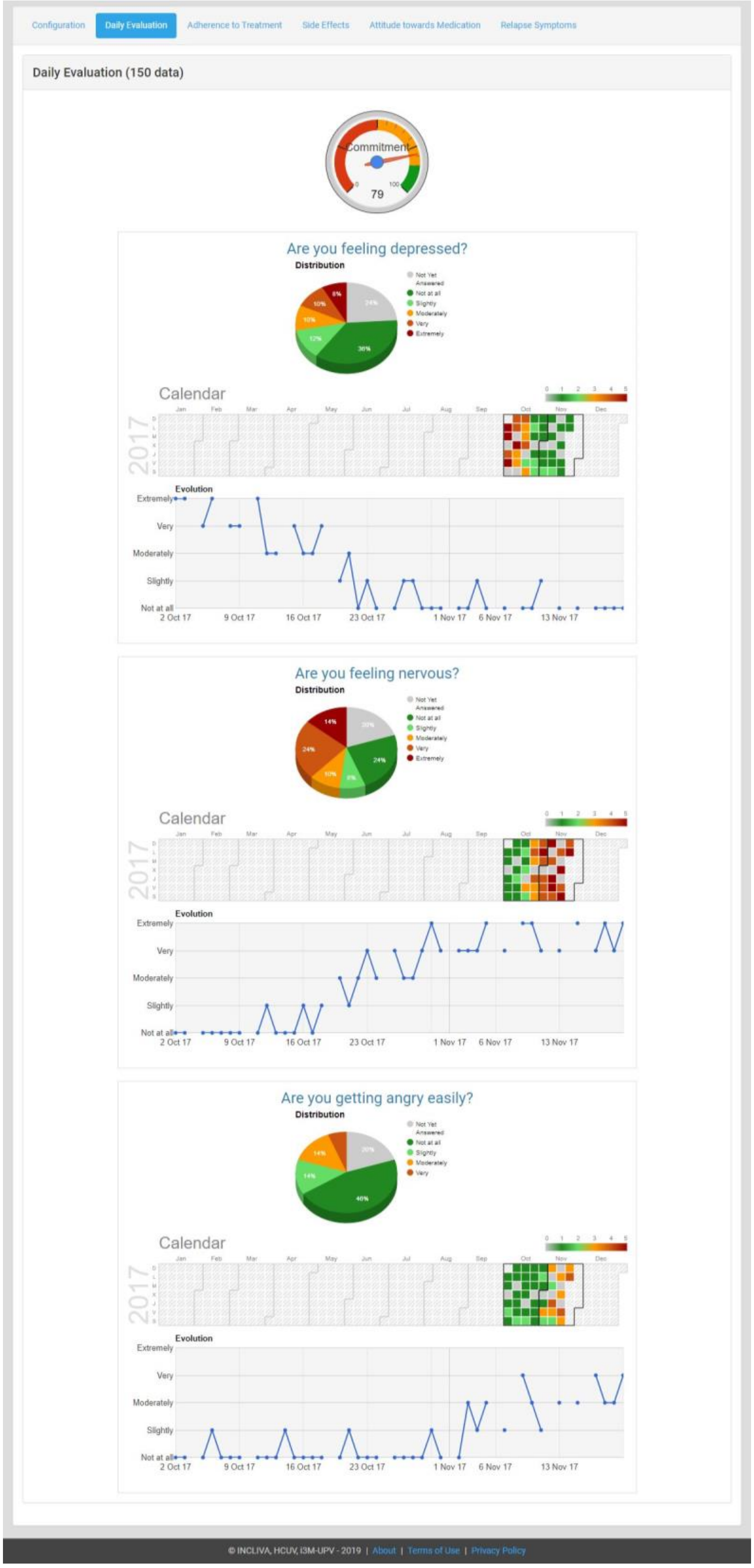

Screenshot of daily assessments from the website.

c. Adherence to treatment tab:

Displays information about daily responses of patients in relation to adherence to antipsychotic medication.

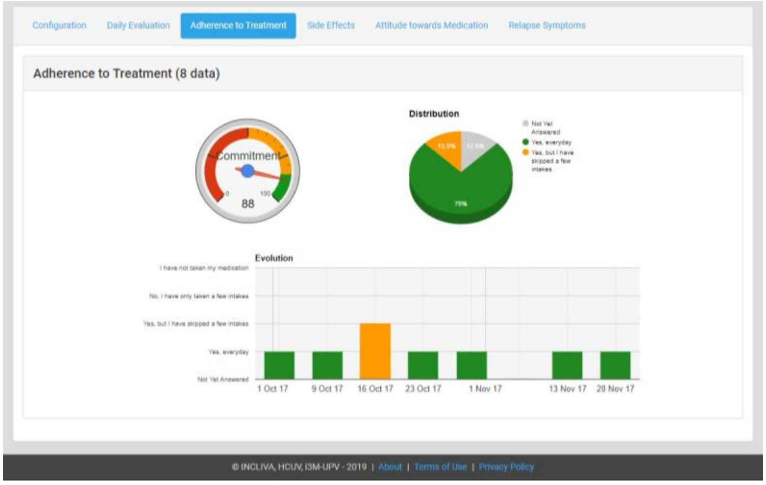

Screenshot of adherence evaluation from the website.

d. Side effects tab:

Displays data related to weekly patient responses in relation to side effects of antipsychotic medication intake.

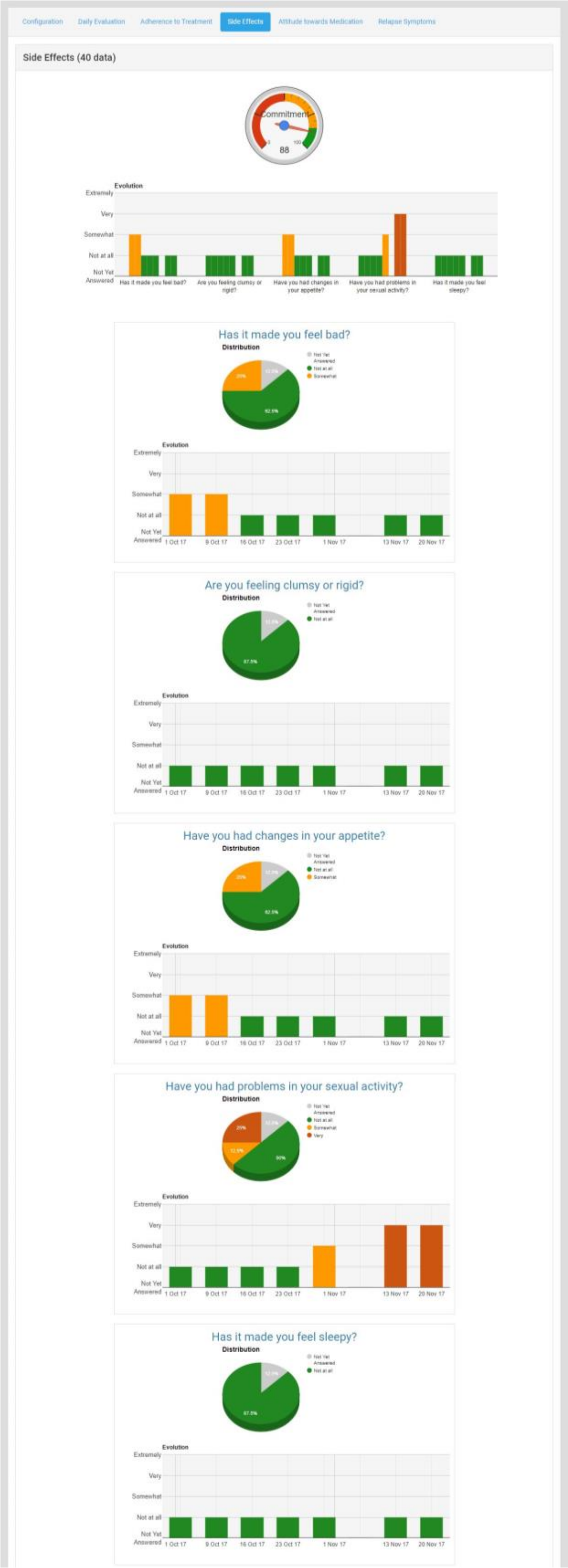

Screenshots of side effect responses displayed on the website.

e. Attitude towards medication tab:

Displays data related to weekly responses of patients in relation to his/her attitude towards antipsychotic medication intake.

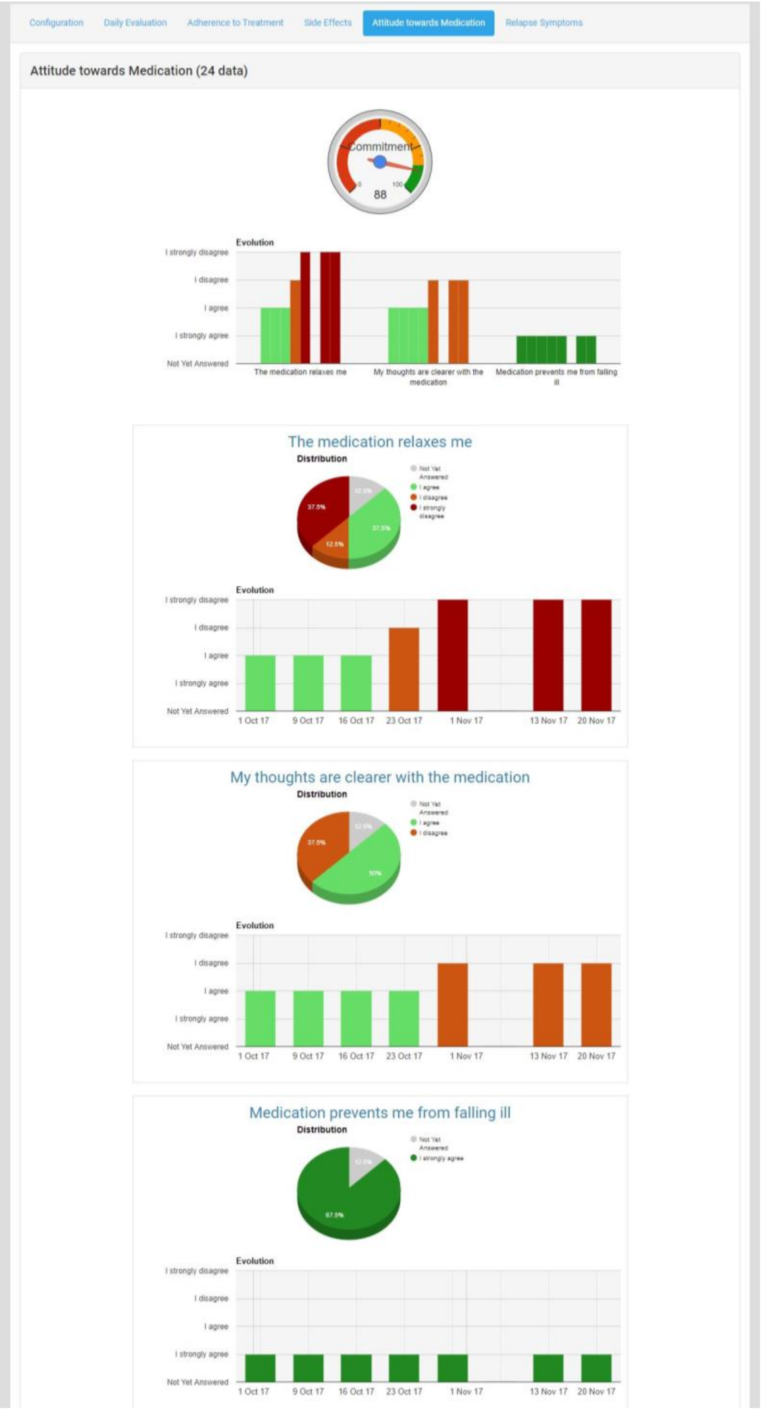

Screenshots of attitude towards medication responses displayed on the website.

f. Relapse symptoms tab:

Displays data to weekly responses of patients aimed to detect a potential relapse into psychotic symptoms.

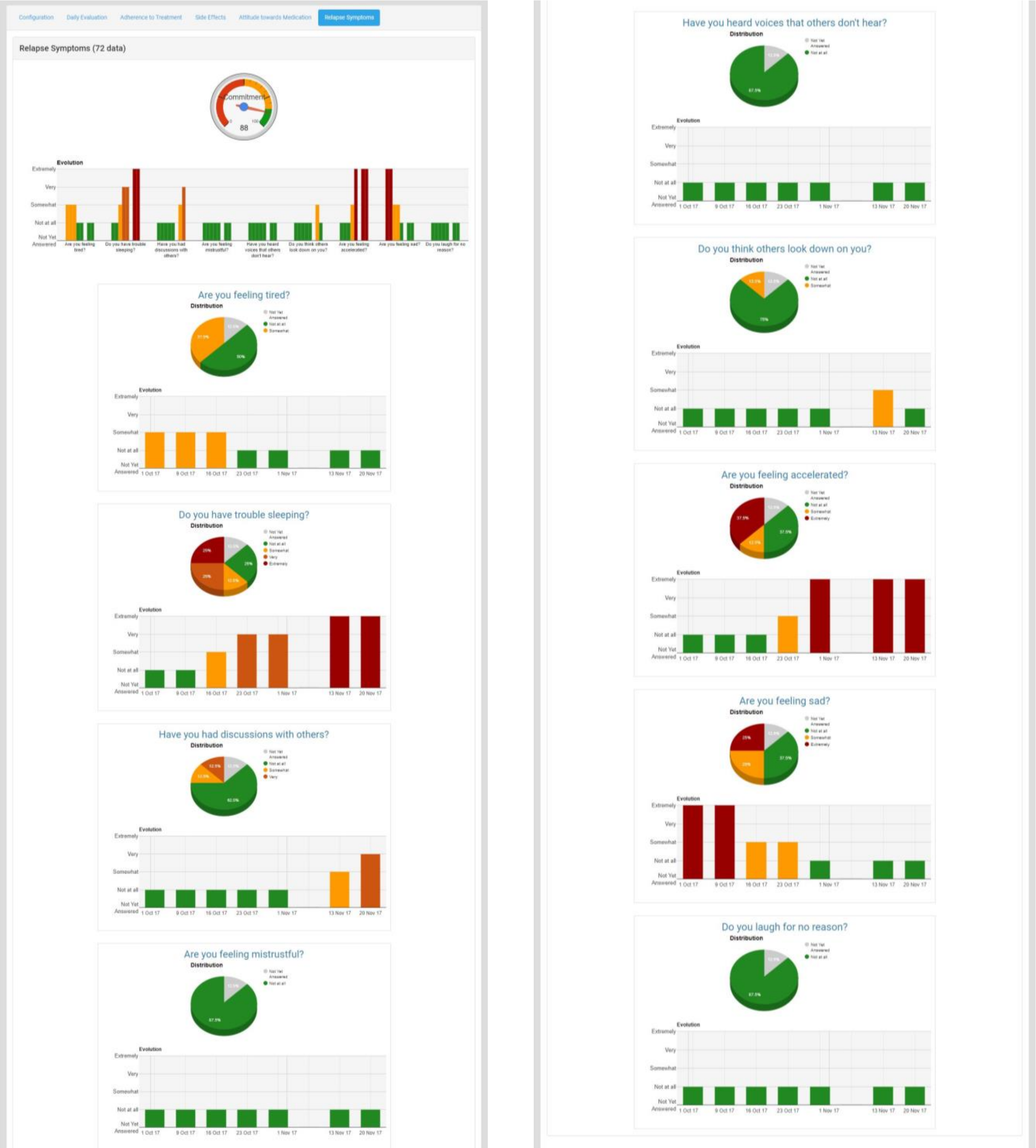

Screenshots of prodromal relapse symptoms responses displayed on the website.

5. APP INSTALLATION INTO PATIENT´S SMARTPHONE:

Once the patient has been registered into the system by the clinician (see Section 3). Registration of the patient into the system), the patient must:

1. Access the profile of the ReMindCare app in the Google Play app store. This can be done by writing the name of the app in the Google Play browser or by accessing this website: https://play.google.com/store/apps/details?id=com.grycap.remindcare

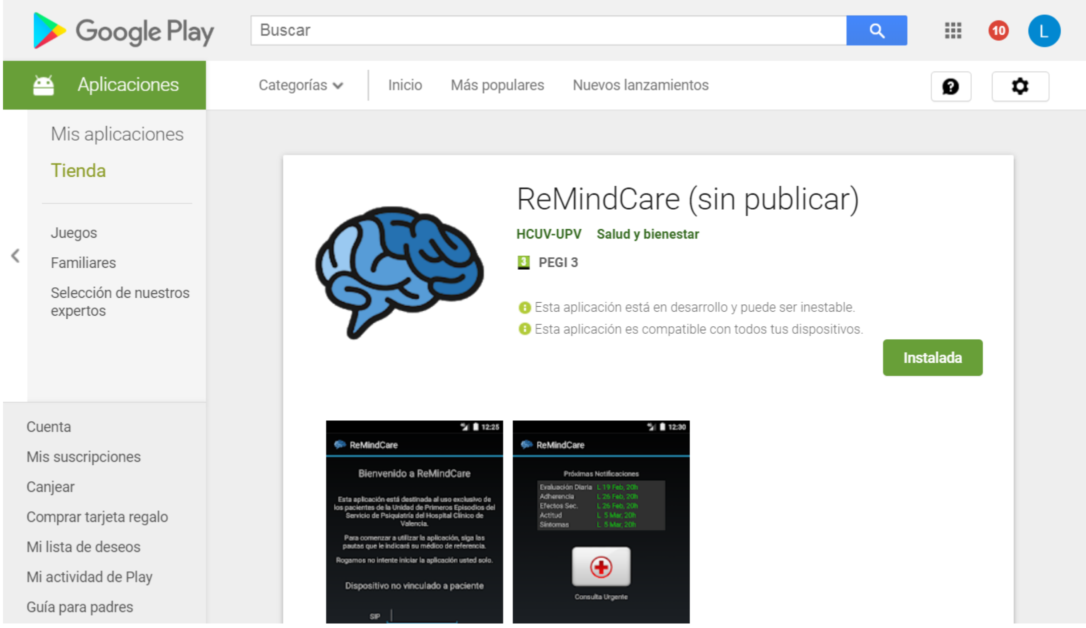

Screenshot of ReMindCare app in Google PlayStore

2. Download the app.
3. Access the app by clicking on it.
4. Type in his or her identification number (SIP) and click the “Start” button.

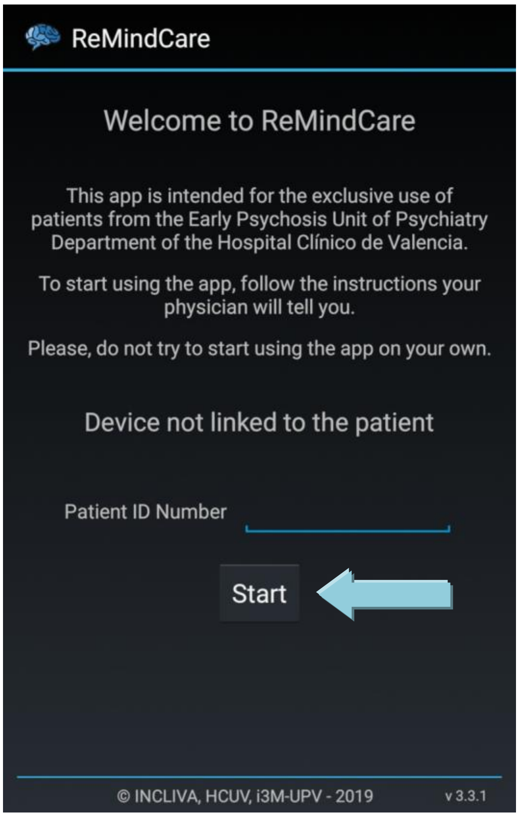

Screenshot of the registration display in the ReMindCare app

6. APP FUNCTIONING:

By clicking the "Start" button, the patient will be registered into the system and will start receiving notifications for daily assessments.

6.1. Notification presentation:

- **Daily assessments:** Every day at 20:00
- **Weekly assessments:** Monday at 20:00

6.2. Response range:

- **Daily assessments:** Questions will be available 6 h from the presentation of the notification.
- **Weekly assessments:** Questions will be available 24 h from the presentation of the notification.

6.3. Questionnaires:

Quick questionnaires aimed to assess the clinical status of the patient. They consist of questions that patients have to answer in accordance with their experiences. Answers are presented on a Likert scale (1 to 5): 1= Not at all, 2= Slightly, 3= Somewhat, 4= Very, 5= Extremely. The questionnaires displayed are the following:

a. Mood status daily evaluation:

It consists of three questions aimed at evaluating his/her mental health status in general.

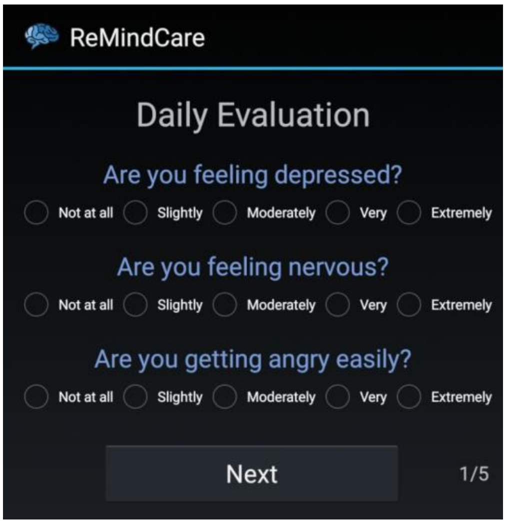

Screenshot of daily evaluation questions in the ReMindCare app.

b. Adherence to medication weekly assessment:

One question regarding adherence to antipsychotic medication.

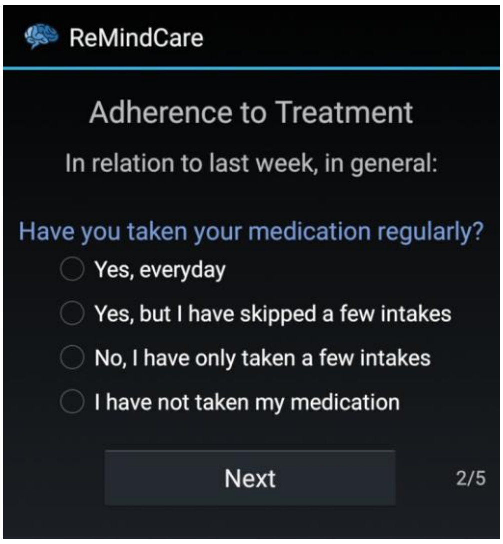

Screenshot of adherence to medication questions in the ReMindCare app.

c. Medication side effects weekly assessment:

Five weekly questions regarding the presence of antipsychotic medication side effects.

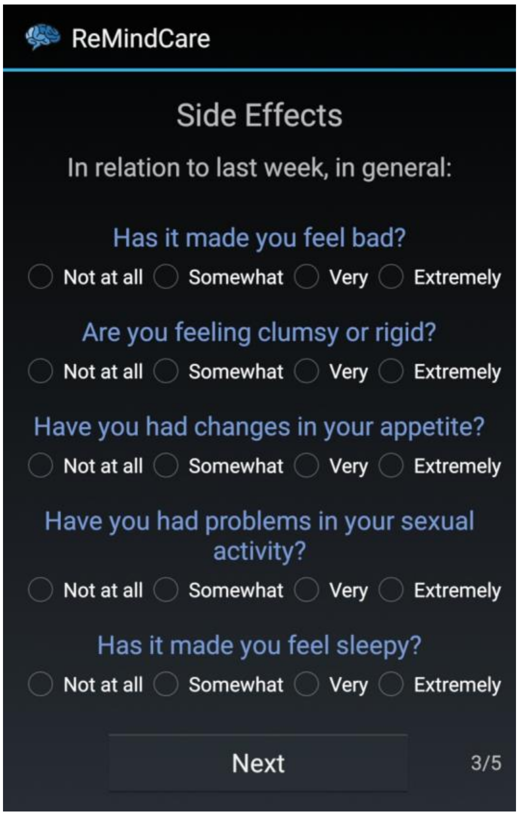

Screenshot of medication side effects questions in the ReMindCare app.

d. Attitude towards medication weekly assessment:

Three questions asked to assess the attitude of the patient towards antipsychotic medication intake.

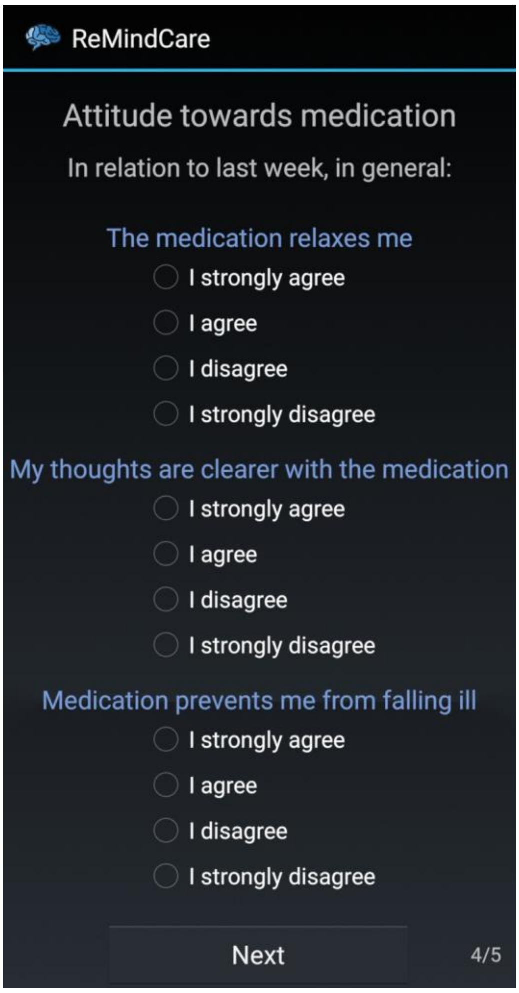

Screenshot of attitude towards medication questions in the ReMindCare app.

e. Prodromal relapse symptoms weekly assessment:

Nine questions aimed to assess the presence of prodromal symptoms of a psychotic relapse.

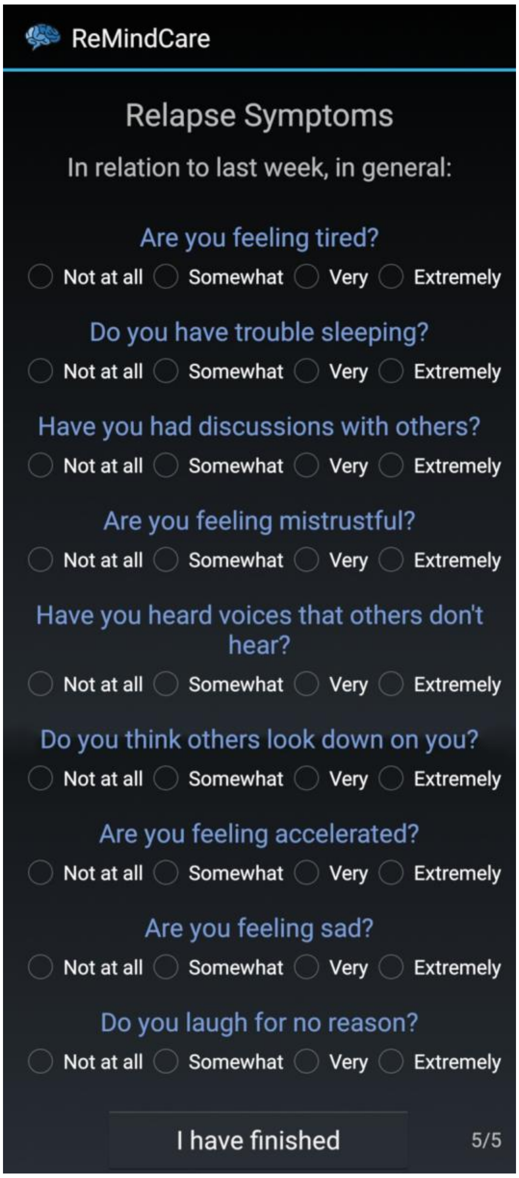

Screenshot of prodromal relapse symptoms questions in the ReMindCare app.

6.4. Alerts system:

There are two types of alerts:

- Alerts automatically generated by the system.
- Alerts deliberately generated by the patient.

a. Alerts automatically generated by the system:

The system can automatically generate three types of alerts related to patient responses to questionnaires presented by the app.

These three types of alerts are as follows:

1. ***Prolonged inactivity:*** This alert appears when the patient has not answered the daily evaluation questionnaires for a period longer than a week, either because he/she has not seen the notifications for questionnaires or because his/her smartphone is switched off or out of service.

2. ***Low compliance:*** This alert appears when the patient has not answered the daily evaluation questionnaires for a period longer than a week because, despite his/her having seen the notifications for the questionnaires; the patient has decided not to answer them.

3. ***Abrupt change:*** This alert appears when the system detects a variation greater than or equal to two points in the patient´s responses to the daily assessment questionnaires or the weekly relapse prodromal symptom assessment.

b. Alerts deliberately generated by patient:

Once the patient logs into the app, he/she has an “Urgent Consultation” tab displayed.

By clicking this button, clinicians ought to contact the patient within a maximum period of 48 hours.

It is important that patients understand that this button must be pushed only when they detect a significant worsening of their clinical status.

However, if a clinician detects an extreme worsening of a patient’s clinical status, the clinician must inform the patient to go to the “Urgent Care Unit” at their hospital of reference immediately.

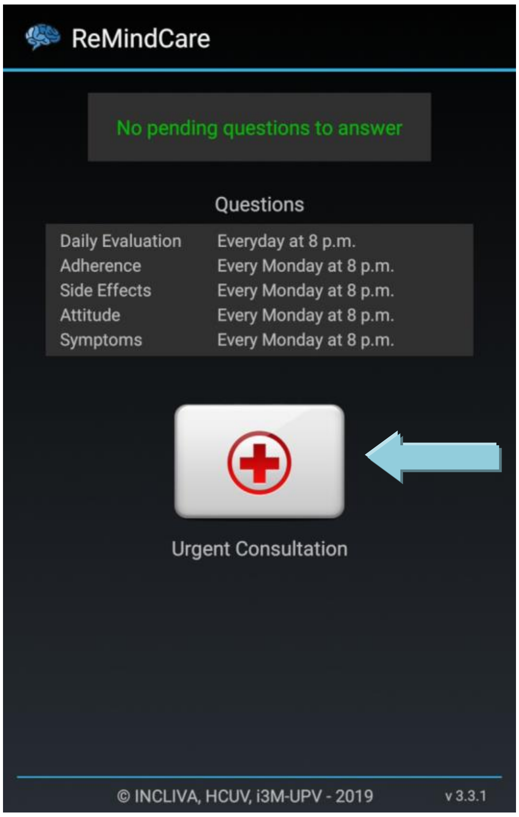

Screenshot of ReMindCare app into patient smartphone.

These alerts generate two types of notifications to the clinician:

1. ***E-mail:*** When the system generates an alert, an automatic notification is sent to clinician's e-mail. In this e-mail, information related to patient identification and characteristics of the alert are given.

2. ***Notification on ReMindCare´s website:*** As shown in *Section 4. Patient data display*, data related to patient alerts are displayed on the main screen and the patient profile page on the website.

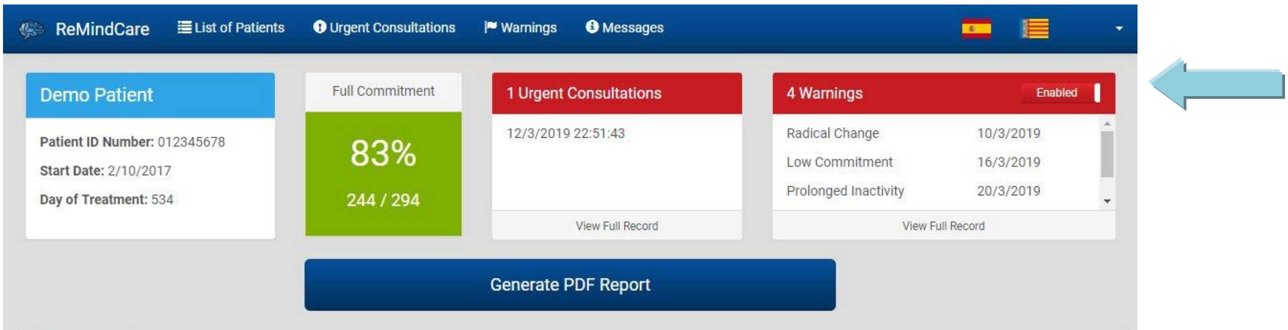

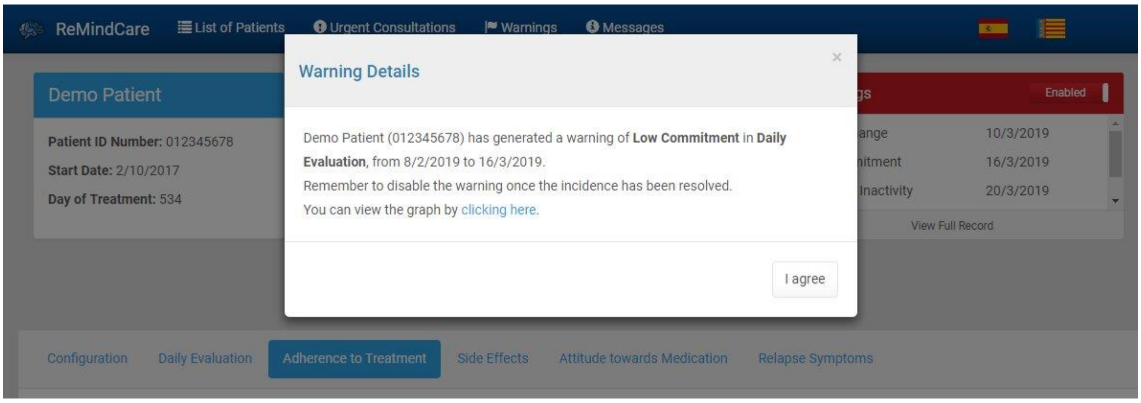

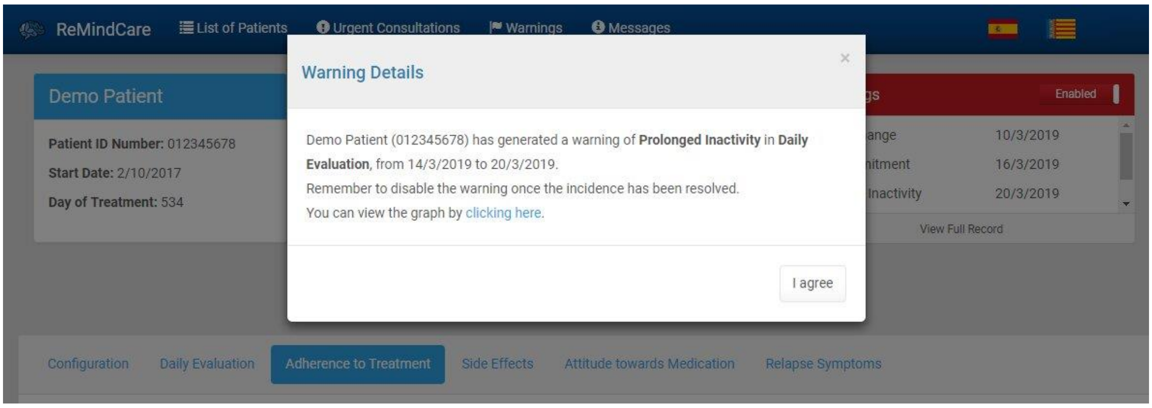

Screenshot sample: Warmings on the ReMindCare website.

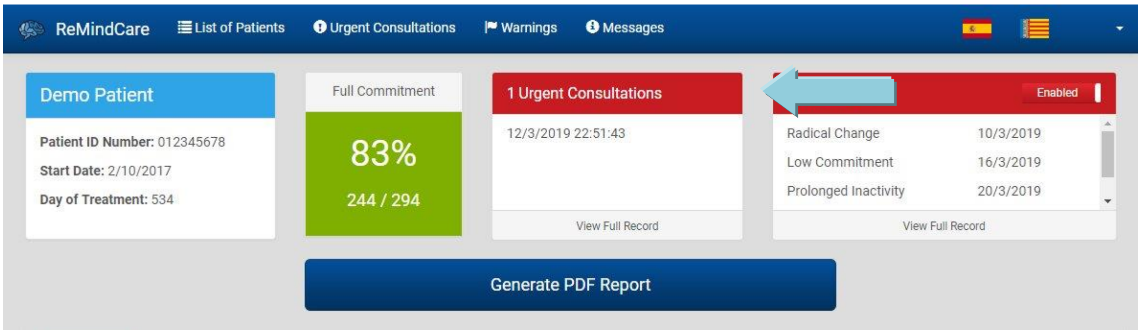

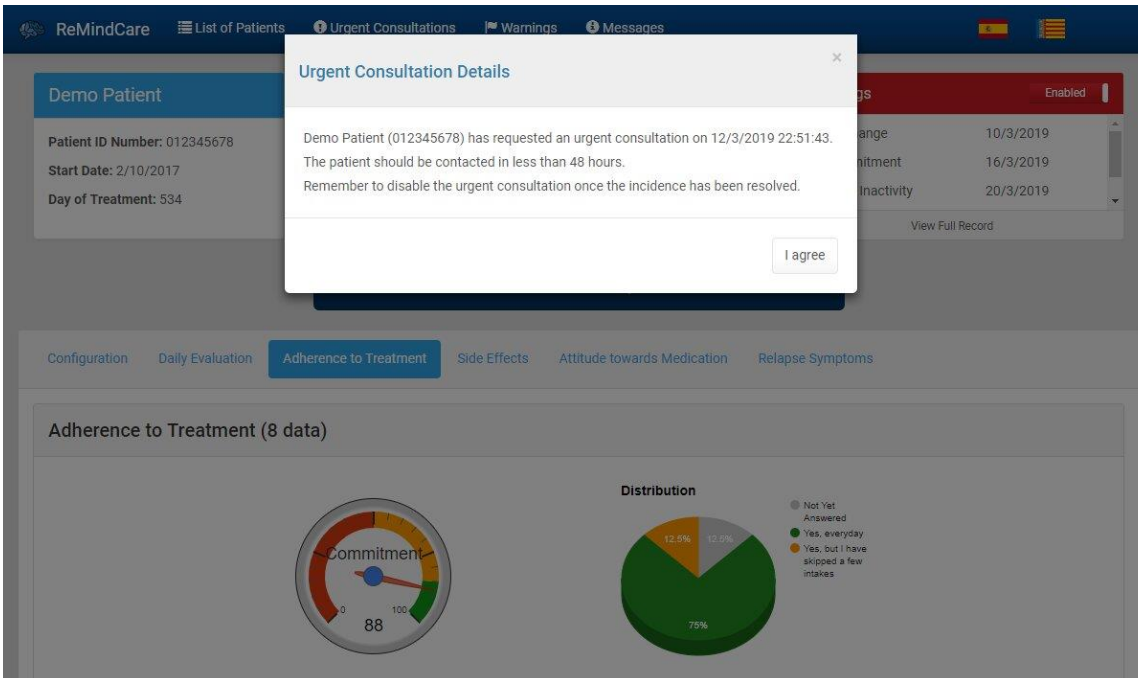

Screenshot sample: Urgent Consultation on the ReMindCare website.

APPENDIX I. PATIENT REPORT SAMPLE

PSYCHIATRY DEPARTMENT
EARLY PSYCHOSIS UNIT

ReMindCare Report

Report Creation Date: 22/03/2019 10:44:55

Patient: [redacted]

Data date range: from 03/10/2018 to 22/03/2019

Full Commitment: 97% (914 of 941 questions answered)

Urgent Consultations generated: 1

Alerts generated: 7

Are you feeling depressed? (Daily Eval.)

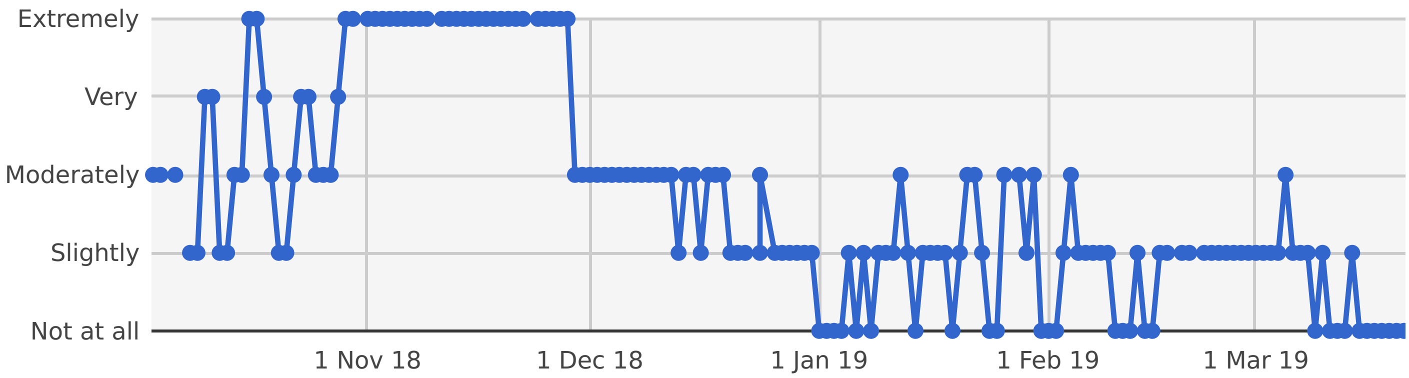

Are you feeling nervous? (Daily Eval.)

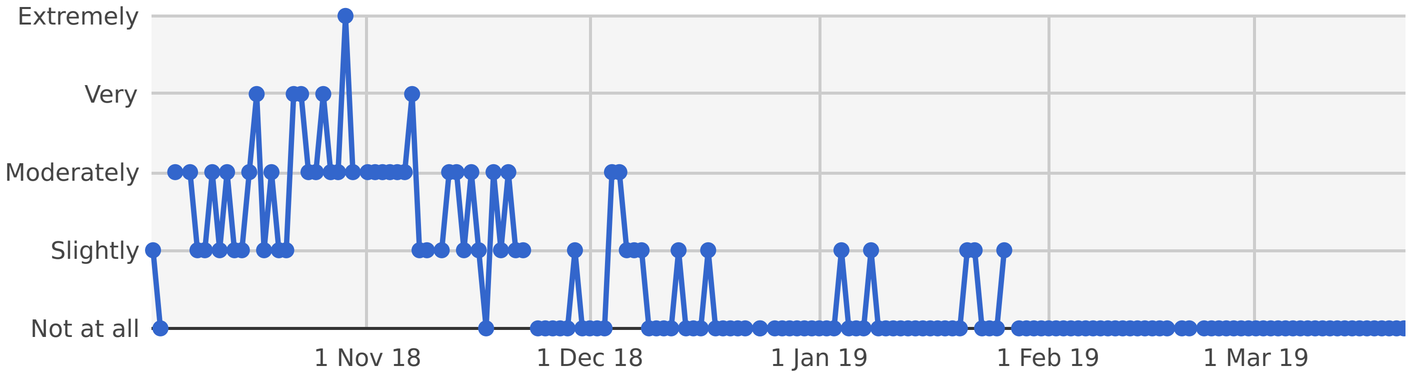

Are you getting angry easily? (Daily Eval.)

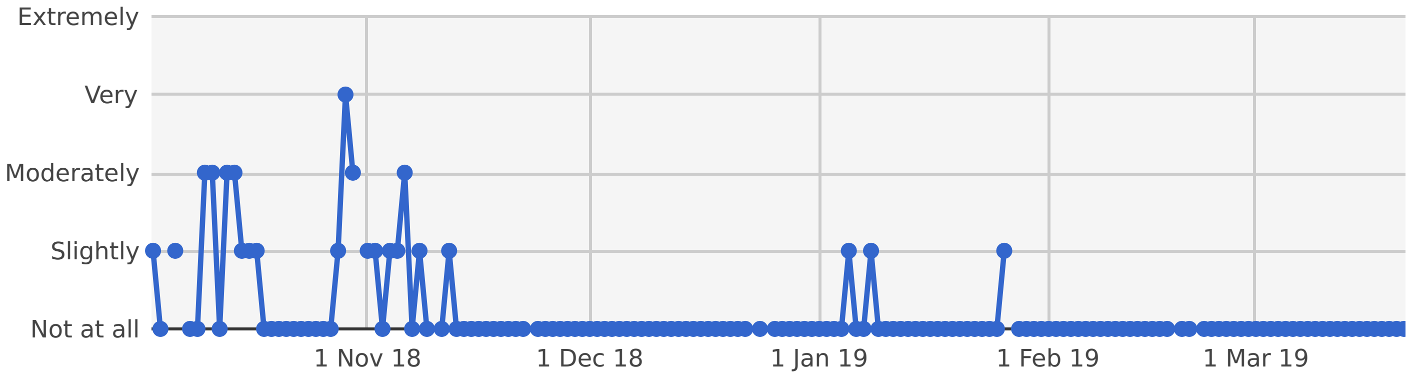

Adherence to Treatment (Weekly Eval.)

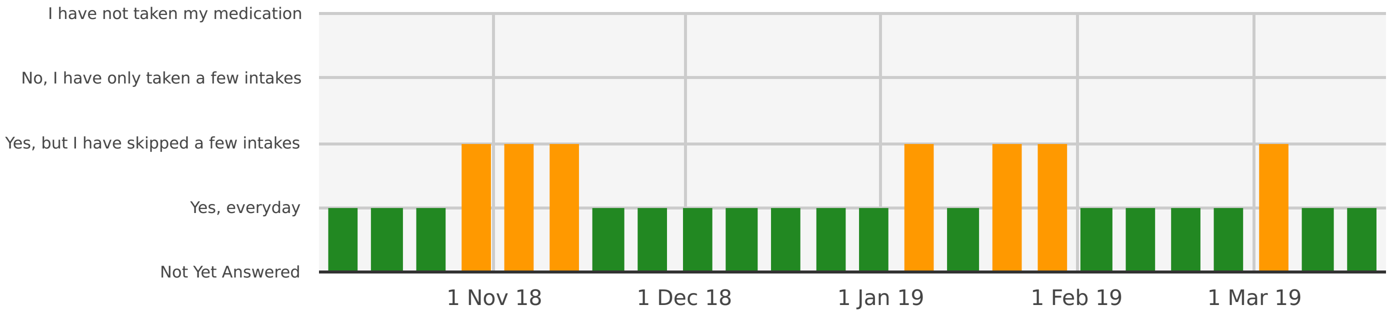

Side Effects (Weekly Eval.)

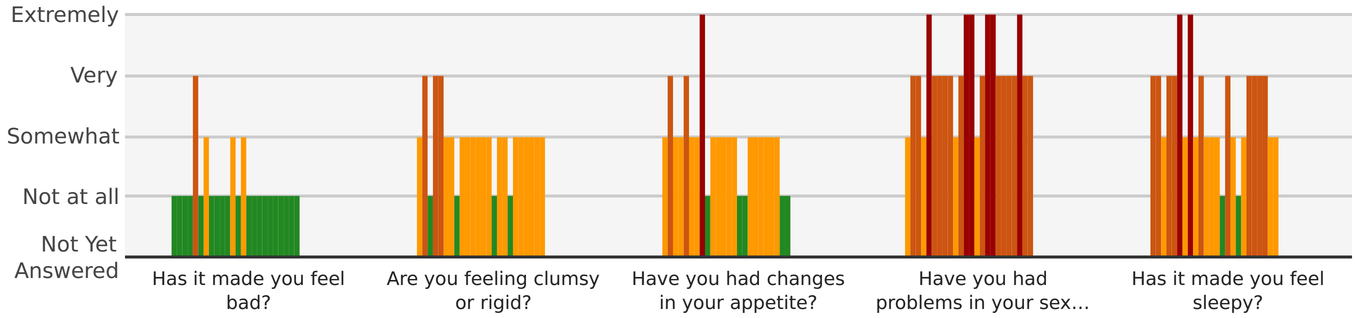

Attitude towards Medication (Weekly Eval.)

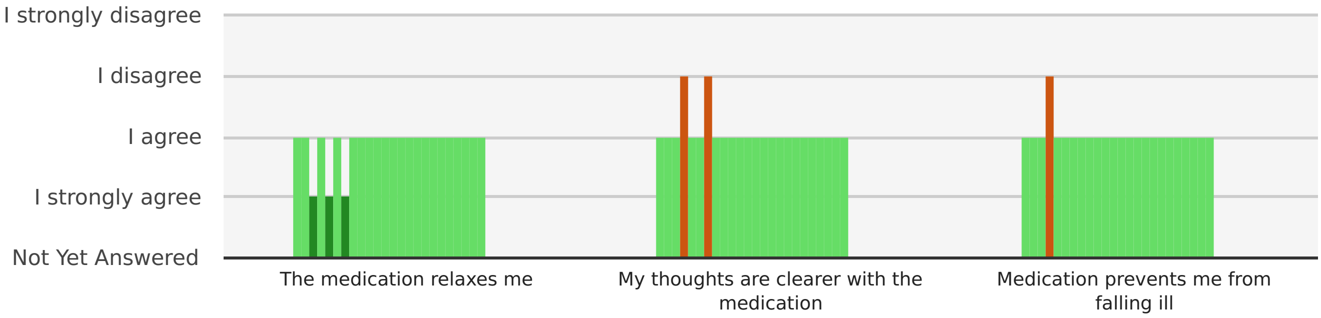

Relapse Symptoms (Weekly Eval.)

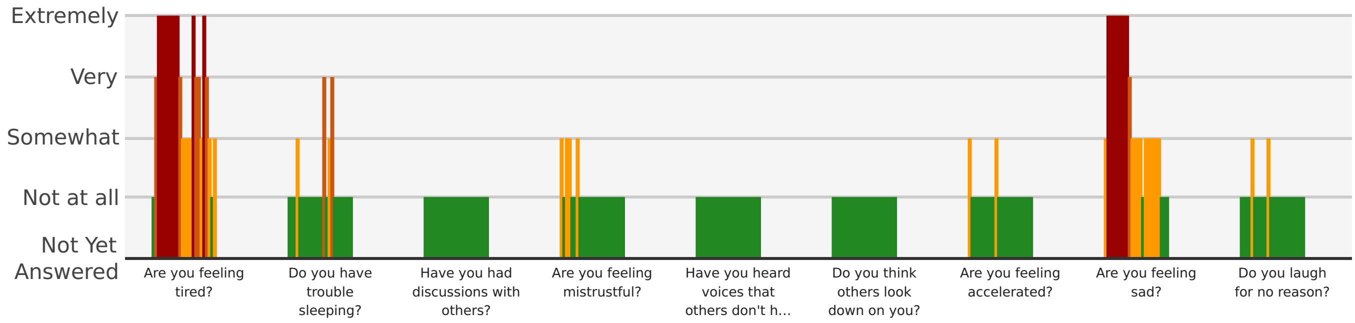

Urgent Consultation History

Day	Time
20/11/2018	16:16:24

Warning History

Warning	From	To
Radical Change en Relapse Symptoms - Are you feeling tired?	08/10/2018	15/10/2018
Radical Change en Relapse Symptoms - Are you feeling sad?	08/10/2018	15/10/2018
Radical Change en Relapse Symptoms - Do you have trouble sleeping?	30/12/2018	07/01/2019
Radical Change en Relapse Symptoms - Do you have trouble sleeping?	06/01/2019	14/01/2019
Radical Change en Relapse Symptoms - Are you feeling tired?	13/01/2019	21/01/2019
Radical Change en Relapse Symptoms - Do you have trouble sleeping?	27/01/2019	04/02/2019
Radical Change en Relapse Symptoms - Are you feeling tired?	11/02/2019	19/02/2019
