# Supplementary material for: ReMindCare, an app for daily clinical practice in patients with first episode psychosis: A pragmatic real‐world study protocol
Source: Early Interv Psychiatry. 2020 Apr 6;15(1):183–92. doi: 10.1111/eip.12960 (PMC7891598; doi:10.1111/eip.12960)
Supplement: Supplementary file 2 — Appendix B. Satisfaction questionnaire [file EIP-15-183-s002.pdf]

REMINDCARE APP FEEDBACK SCALE

VERSION FOR USERS

For the purpose of improving our services, we ask you to fulfil this questionnaire after using ReMindCare app. Please be as sincere as possible, as we would like to know your honest opinion.

This information will be analysed anonymously for research and academic purposes in order to improve quality of healthcare services.

INSTRUCTIONS: *Questions with multiple choice responses will be presented. Cross the box of the response that most accurately describes your opinion.* ***Cross one box per question.***

- *Which is your general opinion after using ReMindCare app? (Advantages, disadvantages, improving aspects...)*

1. **Easy of use.** How easy is learning how to use the app once is installed?

   ☐ Very difficult. It requires a lot of time and effort to learn how to use it.
   ☐ A bit difficult. It took me a bit of time learning how to use it.
   ☐ Easy. After the information clinicians gave me, I knew how to use it.
   ☐ Very easy. Even without the clinician´s help I would have known how to use it.

2. **Performance.** How did the app work? (Notifications, access to test, alert service…)

   ☐ Very poor. I had many problems using the app.
   ☐ Poor. I had some problems using the app but they have been quickly solve or were not very important.
   ☐ Good. Overall functioning of the app is good, but some aspects need to be improved.
   ☐ Very good. App works perfectly, no improvements are needed.

3. **Aesthetics.** Is the visual design of the app appealing?

   ☐ Not attractive at all. Aesthetics need to be improved.
   ☐ Not attractive. Poor design/choice of colours/not appealing.
   ☐ Attractive. I like the overall design it fits with its purpose
   ☐ Very attractive. Programs design is well thought.

B. QUALITY OF INTERVENTION:

1. **Usefulness.** Has the use of the app improved quality of psychiatry healthcare you receive?

   ☐ Not useful at all. Use of the app has not made any changes into my usual psychiatric care.
   ☐ Not very useful. Use of the app has barely improved quality of attention I have received.
   ☐ Useful. I have noticed differences in healthcare attention between using and not using the app.
   ☐ Very useful. Psychiatric attention I received has improved substantively.

2. **Satisfaction.** Are you satisfied with the healthcare service provided by the use of the app?

   ☐ Not satisfied at all. I feel that my responses have not been taken into consideration / clinician has not answer to my notifications…

- ☐ Dissatisfied. I feel that my responses have not been always responded.
- ☐ Satisfied. I feel that my responses have been generally taken into consideration for my clinician as well as my notifications.
- ☐ Very satisfied. I feel that my responses and notifications have been always taken into account.

3. **Recommendation.** Would you recommend this app to other patients with psychosis?

- ☐ No. I would not recommend it.
- ☐ I would just recommend this app to some specific patients but not to all of them.
- ☐ Yes, many patients will benefit from its usage.
- ☐ Yes, it will be beneficial for every patient.

C. USAGE EXPERIENCE:

- *In general, use of the app has made me feel… (Cross the appropriate box):*

	Strongly agree	Agree	Somewhat disagree	Strongly disagree
**Relaxed**. Knowing the app tracked my health status made feel tranquil.	☐	☐	☐	☐
**Anxious**. I feel overwhelming to answer to everyday questionnaires.	☐	☐	☐	☐
**Worried about privacy.** I was worried about information the app was gathering about me.	☐	☐	☐	☐
**Worried about illness.** Use of the app made me think about the illness every day.	☐	☐	☐	☐

	Strongly agree	Agree	Somewhat disagree	Strongly disagree
**Supported.** I felt that my clinician would help me in case I would have felt bad.	☐	☐	☐	☐
**Bored.** Answer daily questions has been very boring.	☐	☐	☐	☐
It has increased **alliance** with my clinician. Thanks to the use of the app I feel that my relationship with my clinician has improved.	☐	☐	☐	☐
